# Identification of sepsis biomarkers through glutamine metabolism-mediated immune regulation: a comprehensive analysis employing mendelian randomization, multi-omics integration, and machine learning

**DOI:** 10.3389/fimmu.2025.1640425

**Published:** 2025-08-20

**Authors:** Zhuang’e Shi, Fuping Wang, Lishun Yang, Couwen Li, Bing Gong, Ruanxian Dai, Guobing Chen

**Affiliations:** ^1^ Faculty of Life Science and Technology, Kunming University of Science and Technology, Department of Emergency Medicine, The Affiliated Hospital of Kunming University of Science and Technology, The First People’s Hospital of Yunnan Province, Medical School, Kunming University of Science and Technology, Kunming, China; ^2^ Kunming Medical University, Department of Emergency Medicine, The First People’s Hospital of Yunnan Province, Kunming, China; ^3^ Medical School, Kunming University of Science and Technology, Department of Emergency Medicine, The First People’s Hospital of Yunnan Province, Kunming, China; ^4^ Department of Emergency Medicine, People’s Hospital of Lijiang, Lijiang, China; ^5^ Department of Emergency Medicine, The First People’s Hospital of Yunnan Province, Kunming, China

**Keywords:** sepsis, mendelian randomization, machine learning, ScRNA-seq, biomarkers

## Abstract

**Background:**

Sepsis is a global health challenge associated with high morbidity and mortality rates. Early diagnosis and treatment are challenging because of the limited understanding of its underlying mechanisms. This study aimed to identify biomarkers of sepsis through an integrated multi-method approach.

**Methods:**

Mendelian randomization (MR) analysis was performed using data on 1400 plasma metabolites, 731 immune cell phenotypes, and sepsis genome-wide association studies. Single-cell RNA sequencing (scRNA-seq) data GSE167363 was used for cell annotation, differential expression analysis, Gene Set Enrichment Analysis (GSEA), transcription factor activity prediction, and cellular pseudotime analysis. The hub genes were identified via least absolute shrinkage and selection operator regression using GSE236713. The predictive models were constructed using the CatBoost, XGBoost, and NGBoost algorithms based on the data from GSE236713 and GSE28750. SHapley Additive ex Planations (SHAP) was used to filter the key molecules, and their expressions were confirmed via RT-qPCR of the peripheral blood mononuclear cells of the patients with sepsis and healthy controls.

**Results:**

Two-step MR revealed that glutamine degradant mediated the causal relationship between SSC-A on HLA-DR + NK and sepsis. ScRNA-seq analysis revealed distinct variations in the composition of immune cell phenotypes in the control and sepsis groups. NK cells were associated with glutamine metabolism. GSEA illustrated the top 10 pathways positively and negatively correlated in NK cells with high vs. low glutamine metabolism. Transcription factor prediction revealed opposing transcription factor profiles for these NK cells subsets. NK cell cellular pseudotime plot and immune cell infiltration analysis results were displayed. The predictive models achieved AUCs of 0.95 (CatBoost), 0.80 (XGBoost), and 0.62 (NGBoost). SHAP analysis identified SRSF7, E2F2, RAB13, and S100A8 as key molecular of the model. RT-qPCR revealed decreased SRSF7 expression and increased RAB13, E2F2, and S100A8 expression in sepsis.

**Conclusion:**

SSC-A on HLA-DR + NK cells reduced the risk of sepsis by decreasing glutamine degradation. SRSF7, E2F2, RAB13, and S100A8 were identified as potential pathogenic biomarkers of sepsis.

## 1 Introduction

In sepsis, an imbalanced immune reaction to pathogens results in dysfunction of multiple organs (1), and it has become a global health problem. Millions of cases are reported globally every year, and approximately one-sixth to one-third of patients die (2–4). For instance, sepsis was diagnosed in one-fifth of patients in an intensive care unit (ICU) in China, and their 90-day mortality rate was 35.5% (5). This has placed a social and economic burden on patients and society. An investigation revealed that patients who had sepsis in the Netherlands had direct and indirect economic costs between 3.8 and 6.5 billion euros (6). The high mortality rate and accompanying burden have been attributed to the unclear pathogenesis and the lack of biomarkers for early diagnosis and treatment.

Multiple studies indicate that sepsis is an immune disorder characterized by excessive inflammatory activation during the early stages and immunosuppression during the later stages (7). The neutrophils, macrophages, natural killer (NK) cells, and monocytes secrete high concentrations of cytokines, leading to a cytokine storm and consequent cell and tissue damage during the development of sepsis (8). The numbers of adaptive immune cells, NK cells, and cytokines and the concentrations of cytotoxic proteins produced by NK cells decrease during the immunosuppressive phase of sepsis (9, 10). Additionally, the function and composition ratio of immune cells also undergo changes, Xue et al. found that the Th2/Th1 ratio was significantly increased in community-acquired severe sepsis and was related to ICU-acquired infection and 28-day mortality (11). The phenotype and effector functions of CD4+ T cells have also been linked to sepsis (12, 13). While several studies investigated the link between immune cells and sepsis, the causal relationship between immune cells phenotype and sepsis and the underlying mechanisms of sepsis have not been fully elucidated. This has posed significant challenges for early diagnosis and treatment of sepsis.

The pathogenesis of sepsis is intricately relationship with plasma metabolite concentrations. Studies have demonstrated the efficacy of sodium butyrate in mitigating sepsis-induced lung injury by modulating immune response and enhancing the barrier functions of the gut and lungs (14). Vitamin K1 has also been implicated in skeletal muscle impairment in sepsis (15). The protective role of gut microbiota metabolites against sepsis-induced intestinal damage has also been reported (16).However, the causal links between metabolites and sepsis, and mediating role of plasma metabolism between immunity and sepsis, and the underlying molecular mechanisms are not fully elucidated. Consequently, we sought to explore causal relationships among immune cells, metabolites, and sepsis, aiming to identify key molecular markers enabling timely sepsis identification and risk assessment.

Mendelian randomization (MR) is a specialized approach, which investigate the causal association between exposure factors and outcomes by eliminating confounding factors (17–20). Although randomized controlled trial (RCT) can also achieve this goal, it’s often difficult to implement. In contrast, MR analysis avoids ethical restrictions and significant funding requirements, enabling the rapid establishment of causal links. Given the widespread use of this method in current causal inference research (21), we propose to employ MR mediation analysis to obtain causal relationship. Multi-omics integration, such as bulk and Single-cell RNA sequencing (scRNA-seq) data analysis, can reveal the molecular mechanisms at the bioinformatics level. While scRNA-seq analyze has been widely employed to uncover molecular mechanisms in sepsis (22), the specific pathways mediating the interplay among immune cells, metabolites, and sepsis remain unclear. Consequently, following the establishment of causal relationships, we will integrate scRNA-seq with bulk data to elucidate these underlying molecular mechanisms. Machine learning (ML) involves training and validating various algorithms on datasets to derive a model with the best configuration for required tasks (23–25). It is widely used in the medical field to analyze big data and can effectively perform tasks across various stages of perioperative anesthetic management, including risk prediction, decision support, and auxiliary diagnostics. Doctors can also gain a comprehensive understanding of patient conditions using insights from the in-depth analysis of big data (26). Some studies have also demonstrated the application of computer-aided drug design and ML in anesthetic drug discovery (27). Given the powerful functionality of ML, we have incorporated it into our study.

Consequently, to elucidate the pathogenesis of sepsis and identify novel makers for diagnosis and targeted therapeutic strategies early, we employed this following integrated approach:(1) MR analysis to delineate causal relationships and mediation effects linking immunity, metabolism, and sepsis; (2) multi-omics integration to characterize underlying molecular mechanisms; (3) Leveraging ML to construct predictive models and discern critical molecules. (4) experimental validation within clinical septic patients. Integrated investigation of multiple methodologies is essential to identify biomarkers for early diagnosis and prediction of sepsis.

## 2 Methods

### 2.1 Study design

**Figure 1:**
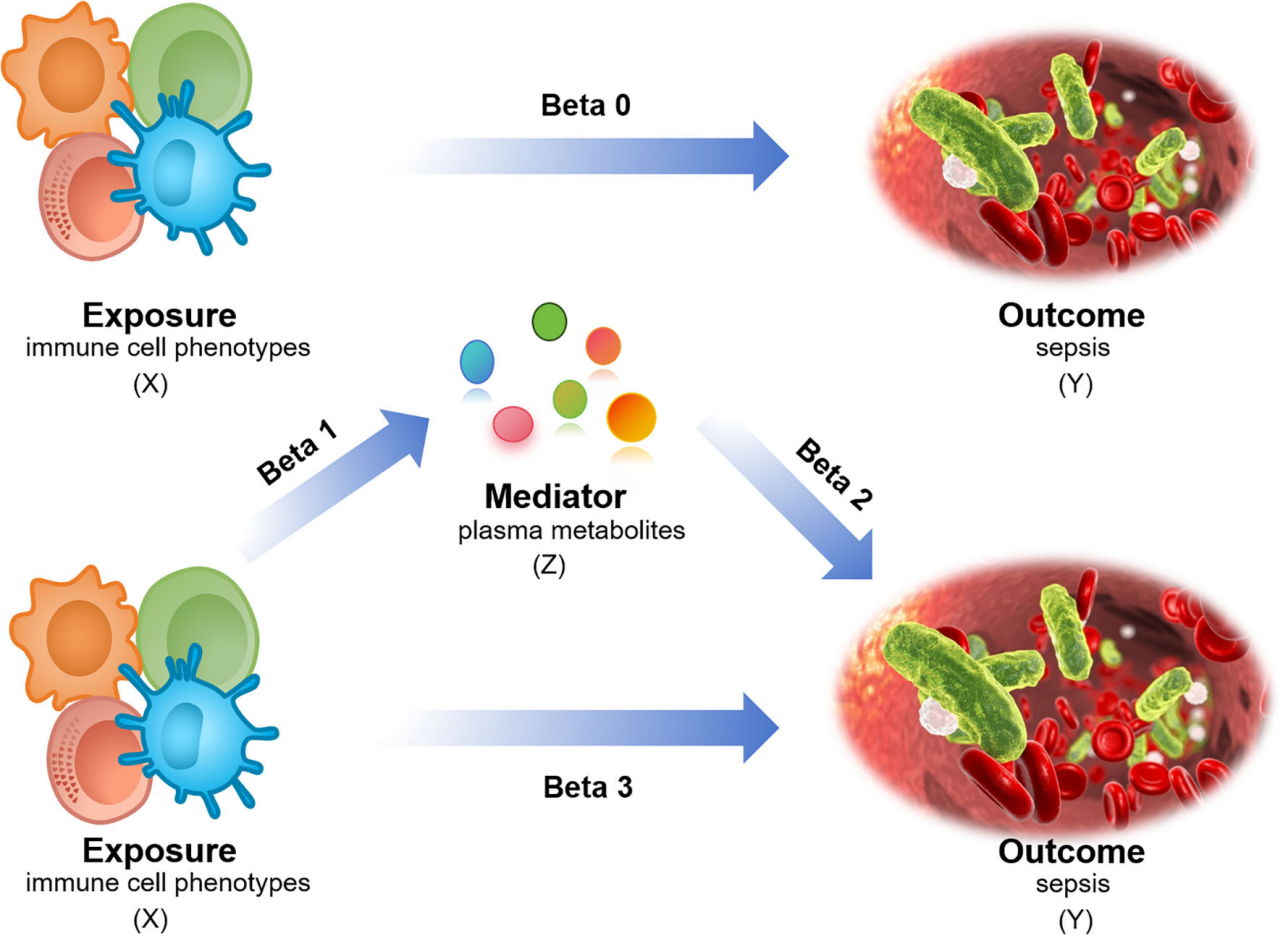
Mediation MR analysis design. X (exposure): immune cell phenotype; Y (outcome): sepsis; Z (mediator): plasma metabolites. Beta0: Total effect; Beta1/Beta2: Mediating/indirect effect; Beta3: Direct effect = total effect - mediating/indirect effect.

**Figure 2:**
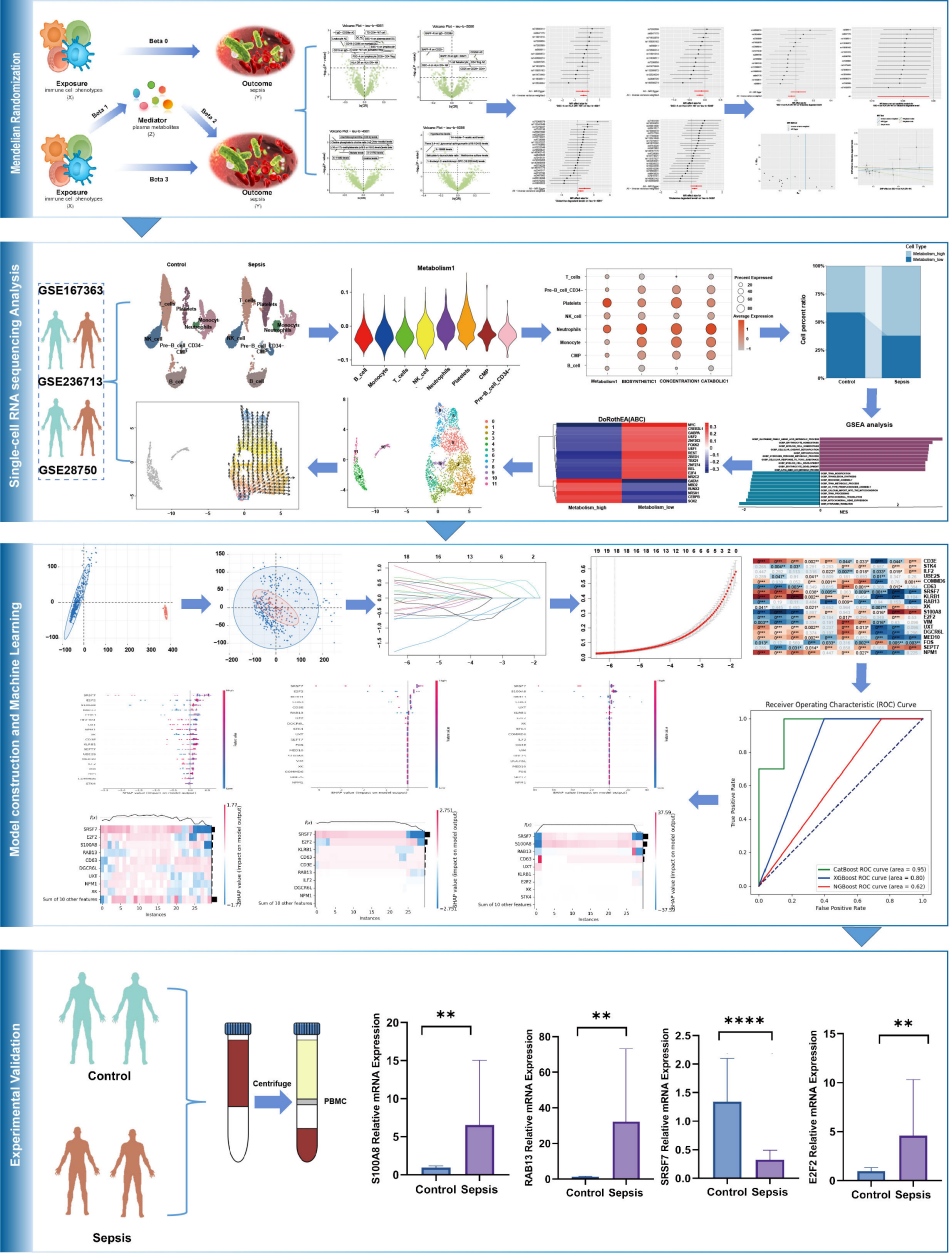
Flowchart and overall design of our study (**:p ≤ 0.01; ****:p ≤ 0.0001).

The causal relationships between 731 immune cell phenotypes and sepsis were investigated using two-sample MR. The 731 immune cell phenotypes were the exposure factors, while sepsis genome-wide association study (GWAS) data were outcome factors. The 1400 plasma metabolites with mediating roles were identified using two-step MR (TSMR) and multivariable MR approaches. The effective instrumental variables (IVs) used in MR met the required assumptions: (1) genetic variation must have a strong direct correlation with exposure factors, (2) genetic factors must not be considered confounding variables in exposure and outcome, (3) genetic polymorphism must not mediate the effects on outcomes through pathways unrelated to exposure (19). The scRNA-seq data were used to obtain immune cell annotations and differentially expressed genes (DEGs), determine cellular timing, and perform Gene Set Enrichment Analysis (GSEA). Additionally, bulk data were analyzed using least absolute shrinkage and selection operator (LASSO) regression to identify hub DEGs. They were subsequently used for immune infiltration and protein-protein interaction (PPI) network analyses. The models for the hub genes were built using CatBoost, XGBoost, and NGBoost methods. Key genes were identified via SHapley Additive explanation (SHAP) analysis, and their expressions were verified via reverse transcription-quantitative real-time polymerase chain reaction (RT-qPCR). The design of the mediation MR analysis is displayed in **Figure 1**, and the complete design of this study is provided in **Figure 2**.

### 2.2 Data source

#### 2.2.1 GWAS data for sepsis

The sepsis GWAS dataset ieu-b-5086 and ieu-b-4981 were downloaded from the GWAS database (https://gwas.mrcieu.ac.uk/) (28), ieu-b-5086 was obtained from the UK Biobank in 2021, which includes 486,484 samples and 12,243,487 single nucleotide polymorphisms (SNPs). The dataset ieu-b-4981, which includes 431,365 samples and 12,243,324 SNPs, was also obtained from the UK Biobank in 2021.

#### 2.2.2 GWAS data for immune cell phenotypes

GWAS data on 731 immune cell phenotypes were obtained from the GWAS catalog (registration numbers: GCST90001391 to GCST90002121). These data were derived from a cohort study involving 3,757 individuals with European ancestry in 2020 (29). The 731 immune cell phenotypes included 118 absolute cell counts (ACs), 389 median fluorescence intensities reflecting surface antigen levels (MFI), 32 morphological parameters (MP), and 192 relative cell counts (RC) (30).

#### 2.2.3 GWAS data for plasma metabolites

Plasma metabolite GWAS data (GCST90199621-GCST90201020) were sourced from the GWAS catalog, originating from the Canadian Longitudinal Aging Study (n=8,299) (31).

#### 2.2.4 Bulk and scRNA-seq datasets

The scRNA-seq datasets for GSE167363 were obtained from the Gene Expression Omnibus (GEO) database(https://www.ncbi.nlm.nih.gov/geo/), and the scRNA-seq of human peripheral blood mononuclear cells (PBMCs) from healthy controls, survivor and non-survivor of gram-negative sepsis patients, control samples GSM5102900, GSM5102901 and sepsis samples GSM5102903 and GSM5511352 were chosen. The GSE236713 and GSE28750 datasets were obtained from GEO database and used for model development.

### 2.3 Pathway enrichment data

The GMT files for Kyoto Encyclopedia of Genes and Genomes (KEGG) glutamine metabolism, glutamine biosynthesis, glutamine concentration, and glutamine catabolism were downloaded from the MSigDB (https://www.gsea-msigdb.org/gsea/msigdb).

### 2.4 IVs selection

The IVs were significantly correlated with the exposure factors but independent of the outcomes. SNPs significantly associated with the exposures were identified to serve as IVs. The significance level was set at P < 1 × 10^-5^. Linkage disequilibrium (LD) clumping was performed using the 1000 Genomes Project Phase 3 European reference panel with stringent parameters: an LD threshold of r² < 0.001 and a window size of 10,000 kb. This ensured independence of instrumental variables while accounting for population-specific genetic architecture (32, 33). SNP selection required F-statistics >10, with PhenoScanner used to remove variants potentially affected by confounding (34).

### 2.5 MR analysis

Mediation MR analysis was performed using the TwoSampleMR package in R version 4.4.1. The inverse variance weighting (IVW) method was employed. Two-sample MR analysis was used to explore the causal relationship between 731 immune cell phenotypes and ieu-b-5086, ieu-b-4981 sets, 1400 plasma metabolites and ieu-b-5086, ieu-b-4981 sets. Immune cell phenotypes and metabolites with causal relationship between the two datasets were selected. Second, metabolites were analyzed as mediators using TSMR.

### 2.6 ScRNA-seq data analysis

The scRNA-seq data were analyzed using the “Seurat” R package. GSM5102900 and GSM5102901 were grouped as controls and GSM5102903 and GSM5511352 were grouped as sepsis controls. We performed data quality control by removing mitochondrial, ribosomal, and red blood cell genes, this ensured that mitochondrial gene content <10% and detected gene count: 200-4000. “NormalizeData” R function was used to data standardized. Dimensionality reduction and clustering based on principal component analysis (PCA) was carried out with the “tidyverse” and “patchwork” R packages. Batch effects between the samples were removed using the “Harmony” R package. Further dimensionality reduction, clustering, and visualization were performed using the “UMAP” R package. The cell clusters were visualized and annotated using the “SingleR” R package. The “read.gm” and “AddModuleScore” functions were applied to incorporate the KEGG pathway scores of immune cells.

### 2.7 GSEA analysis

GSEA represents a robust computational approach to functionally interpret gene sets through expression profiling (35). GSEA was performed using “ClusterProfiler” R package, and the top10 pathways were visualized.

### 2.8 Transcription factor activity prediction and cellular pseudotime analysis

As critical regulators of transcription, TFs bind to specific DNA sequences to control the activation or repression of target genes, and they influence gene expression within the cell. TF activity prediction facilitates the exploration of the molecular mechanisms of diseases (36), it was forecasted using the “Dorothea” R package.

The trajectory of cell development was identified based on the expressed genes via cellular pseudotime analysis. The “Vector” function was used for cellular pseudotime analysis.

### 2.9 Screening hub genes

GSE236713 was chosen as the training dataset, GSE28750 was chosen as the test dataset. GPL17077–17467 and GPL570–55999 were used or gene ID conversion. The “limma” R package was employed to identify DEGs. Statistical significance was set at p < 0.05 and logFC > 1. LASSO regression was performed to identify the hub genes after removing the batch effects and PCA analysis. Immune cell infiltration was analyzed using “IOBR” R package.

### 2.10 Machine learning

CatBoost, XGBoost, and NGBoost ML algorithms to establish the model. These algorithms are favored for their adaptability, scalability, and user-friendliness and have been widely applied to various research domains (37–39). The CatBoost model parameters are configured as: 1000 iterations, depth of 6, L2 leaf regularization of 3.0, learning rate of 0.03, with “Logloss” as the loss function and “Accuracy” as the evaluation metric. For the NGBoost model, the parameter setup includes: Dist set to k categorical (2), Score using LogScore, 1000 estimators, minibatch fraction of 1.0, column sample of 1.0, learning rate of 0.01, verbose evaluation every 100 rounds, Base as the default tree learner, and a tolerance of 0.0001. Regarding the XGBoost model, its parameters consist of: 100 estimators, subsample ratio of 1.0, learning rate of 0.3, colsample_bytree of 1.0, maximum depth of 6, reg_lambda of 1.0, and the objective function specified as ‘binary: logistic’. To analyze the molecular contribution mechanism of the CatBoost, XGBoost, and NGboost models. SHAP is a model interpretation method based on the cooperative game theory. It provides global feature importance and interpretability for individual predictions by calculating the marginal contribution (Shapley value) of each feature. The core advantage is its adherence to the consistency principle: an increase in the dependence of the model on a feature is accompanied by an increase in the importance score assigned by SHAP. This helps avoid the ranking bias associated with differences in the model structure in traditional feature importance methods. We used the SHAP framework to assess the feature importance and analyze the molecular contribution mechanisms of the CatBoost, XGBoost, and NGBoost models. The SHAP values for all test set samples were calculated using the Python SHAP library (v0.42.1). The average absolute SHAP value of each molecular feature was visualized using a SHAP summary plot for each model, and the top three key molecules of global importance were identified based on this.

### 2.11 Peripheral blood mononuclear cells

Ethical approval was obtained from the Research Ethics Committee of the First People’s Hospital of Yunnan (Approval number: KHLL2025-KY021). All experimental procedures adhered to applicable named guidelines and regulatory requirements, with informed consent secured from all participants and/or their legal guardians. Twenty patients with sepsis and 20 healthy volunteers were selected from Yunnan First People’s Hospital. The PBMC were extracted using a lymphocyte isolation solution (Solarbio Life Sciences, Beijing, China) and red blood cell lysates (Solarbio Life Sciences, Beijing, China).

### 2.12 RT-qPCR and statistical analysis

Total RNA was isolated from the human PBMCs using TRIzol reagent (Invitrogen, Carlsbad, CA, United States). NanoDrop (Thermo fisher Scientific, number: ND-ONEC-W) was used to evaluate RNA purification and measure the concentration. RNA was reverse transcribed into cDNA using the Evo M-MLV RT Kit (Accurate Biotechnology [Hunan] Co. Ltd.). The RT-qPCR system was established using the SYBR Green Premix Pro Taq HS qPCR Kit (Accurate Biology) and a fluorescence quantitative polymerase chain reaction instrument (CFX96TM; Bio-Rad, United States). GAPDH was used as the reference gene. Sangon Biotech (Shanghai) Co., Ltd. synthesized the primer sequences (**Table 1**). Their levels of expression was calculated using relative quantification (2^−ΔΔt^ method) (40). The Wilcoxon test was employed to assess the correlation between two groups, and the Kruskal-Wallis test utilized to examine the relationship among three groups. A significance level of P < 0.05 was adopted for statistical evaluation.

**Table 1:** The synthesized the primer sequences.

Gene	Primer sequences
SRSF7	Forward:5’- TGCTATGAGTGTGGCGAAAAGGG -3’ Reverse: 5’- GACCGTGACCTGCTTCTTCTTCG -3’
E2F2	Forward:5’- CCCGTCGTCCCTGAGTTCCC -3’ Reverse: 5’- CCAGCGAAGTGTCATACCGAGTC -3’
RAB13	Forward:5’- GTCAGGAGGCCGGAGATCAGG -3’ Reverse: 5’- CAGCCCAGGGAGCACTTGTTG -3’
S100A8	Forward:5’- TGCTAGAGACCGAGTGTCCTCAG -3’ Reverse: 5’- GCCACGCCCATCTTTATCACCAG -3’

## 3 Result

### 3.1 Causal links between five immune phenotypes and sepsis

**Figure 3:**
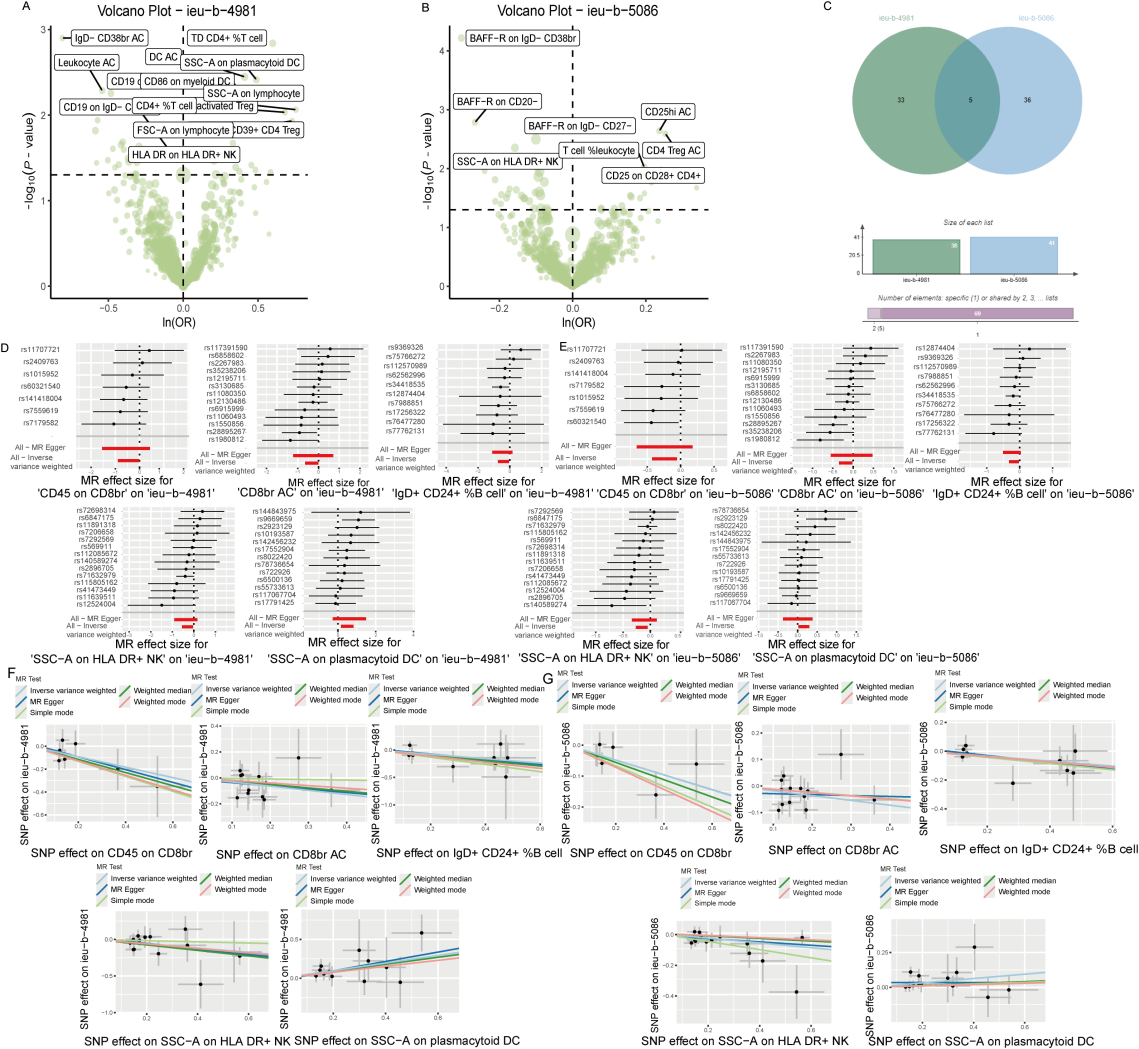
The causal relationship between immune phenotype and sepsis. **(A)** Volcano plot of the causal relationships between the immune phenotypes and the ieu-b-4981 dataset. **(B)** Volcano plot of the causal relationship between the immune phenotype and the ieu-b-5086 dataset. **(C)** Venn diagram of the five immune phenotypes in the ieu-b-4981 and ieu-b-5086 datasets. **(D, E)** Forest plots of the relationships between five immune cell phenotypes and sepsis based on the ieu-b-4981 **(D)** and ieu-b-5086 dataset **(E)**. **(F, G)** Scatter plots showing the causal relationships between five immune cell phenotypes and sepsis based on the ieu-b-4981 **(F)** and ieu-b-5086 datasets **(G)**.

Analysis of the causal link between the immune phenotype and sepsis. The GWAS ieu-b-4981 dataset analyzed using the IVW method revealed 38 immune phenotypes that were causally related to sepsis (p < 0.05). A volcano plot is shown in **Figure 3A**, and detailed data are shown in **Supplementary Table S1**. Analysis of the GWAS ieu-b-5086 dataset using the IVW method revealed 41 immune phenotypes causally related to sepsis (p < 0.05). A volcano plot is shown in **Figure 3B**, and detailed data are presented in **Supplementary Table S2**. The intersection were five immune phenotypes which were causal relationship with sepsis, there were CD45 on CD8br, CD8br AC, IgD+ CD24+ %B cell and SSC-A on HLA DR+ NK and SSC-A on plasmacytoid DC respectively, the Venn diagram was presented in **Figure 3C**. Forest plots revealed that SSC-A on HLA DR+ NK, CD45 on CD8br, CD8br AC, and IgD+ CD24+ %B cell could reduce the risk of sepsis (IVW, b < 0), whereas SSC-A on plasmacytoid DC increase the risk of sepsis (IVW, b > 0). The casual relation between immune phenotype and sepsis were calculated via five methods (IVW, weighted median, MR egger, weighted mode, and simple mode), and the results were visualized using scatter plots shown in **Figures 3D, E**. The results revealed that the greater of SNP effect on CD45 on CD8br, CD8br AC, IgD+ CD24+ %B cell and SSC-A on HLA DR+ NK were associated with a lower risk of sepsis, Conversely, greater effects of SNPs on SSC-A in plasmacytoid DCs were associated with a higher risk of sepsis, the result shown in the **Figures 3F, G**. All data underwent bias assessments and leave-one-out sensitivity analyses to enhance the robustness of the MR results. The funnel plot and leave-one-out plot are provided in **Supplementary Figure S1**.

### 3.2 Causal links between 13 plasma metabolites and sepsis

**Figure 4:**
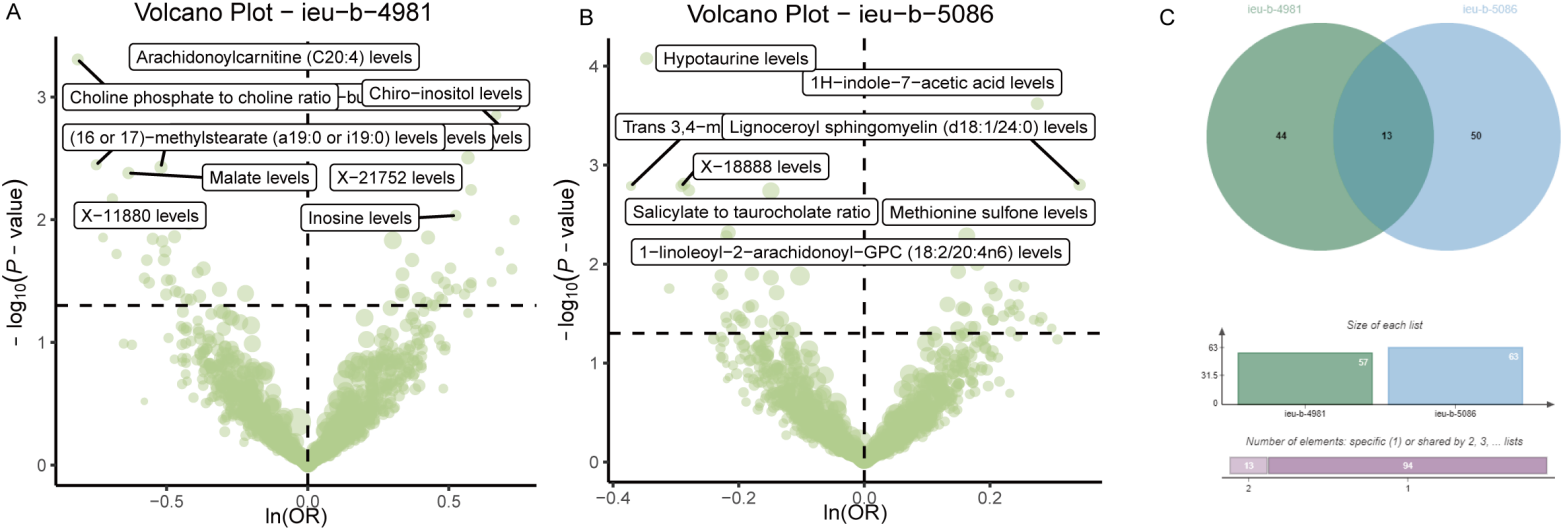
Causal relationship between plasma metabolites and sepsis. **(A)** Volcano plot of the causal relationships between plasma metabolites and the ieu-b-4981 dataset. **(B)** The volcano plot of the causal relationships between plasma metabolites and the ieu-b-5086 dataset. **(C)** Venn diagram of the 13 plasma metabolites in the ieu-b-4981 and ieu-b-5086 datasets.

**Figure 5:**
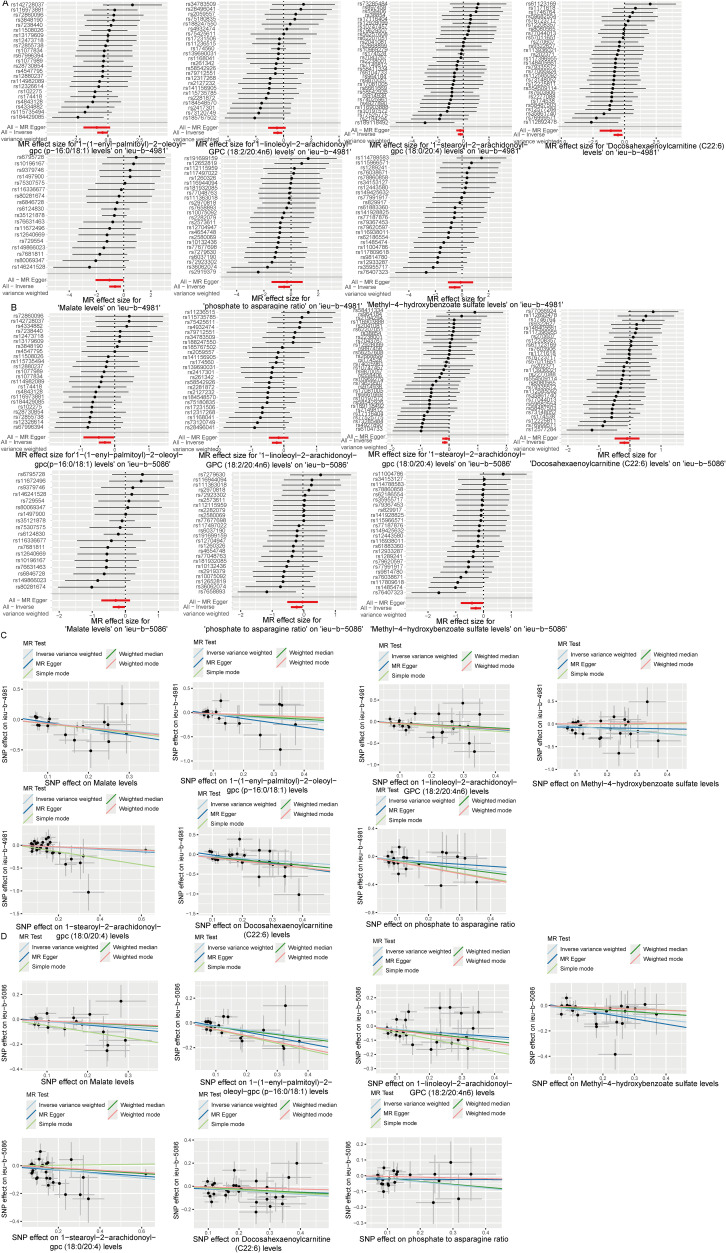
Six plasma metabolites associated with an increased risk of sepsis. **(A, B)** Forest plots of the causal relationships among the six positive plasma metabolites and sepsis based on the ieu-b-4981 **(A)** and ieu-b-5086 **(B)** datasets. **(C, D)** Scatter plots of the causal relationships among the six positive plasma metabolites and sepsis based on the ieu-b-4981 **(C)** and ieu-b-5086 **(D)** datasets.

**Figure 6:**
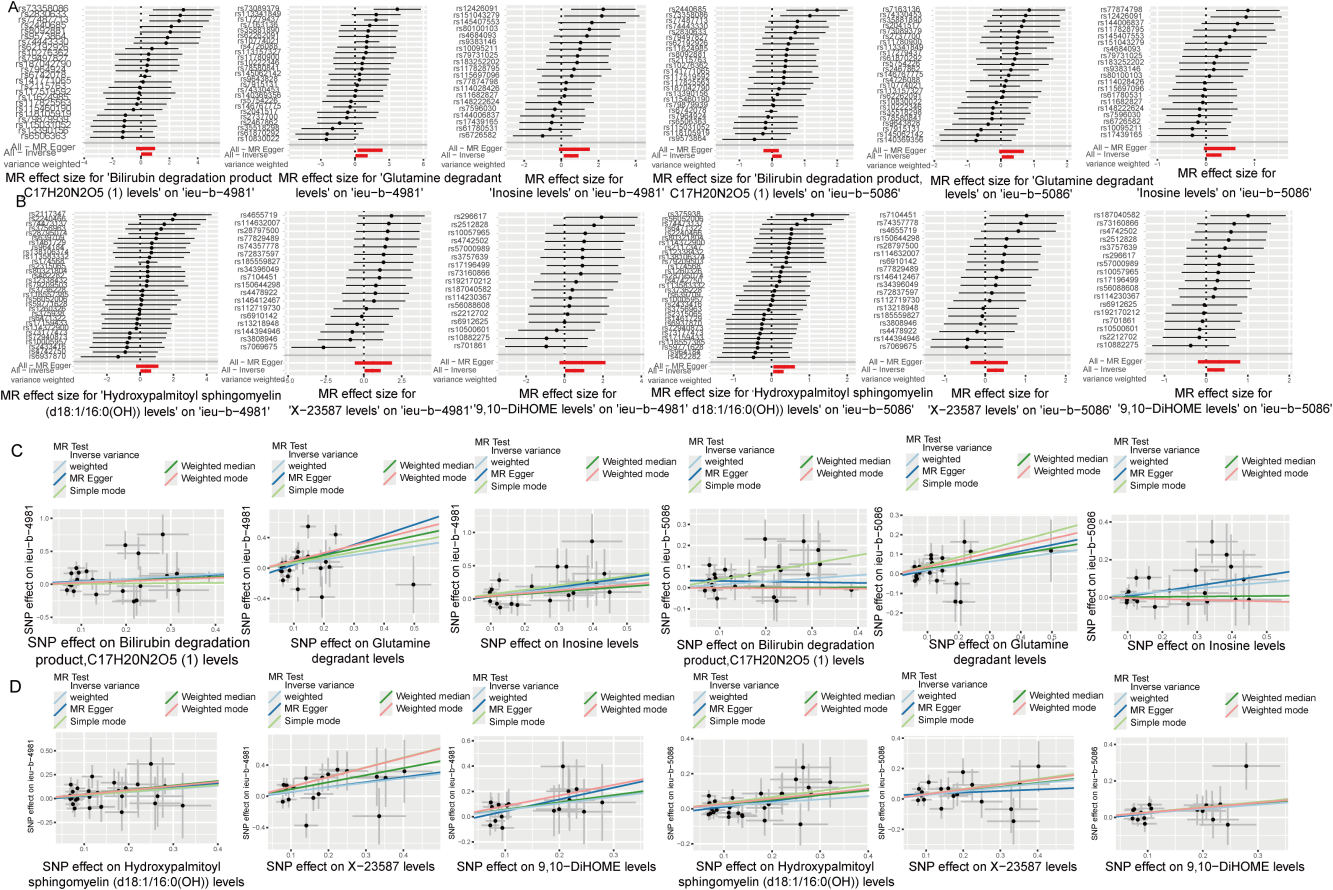
Seven plasma metabolites associated with a reduced risk of sepsis. **(A, B)** Forest plots of the causal relationships among seven negative plasma metabolites and sepsis based on the ieu-b-4981 **(A)** and ieu-b-5086 **(B)** datasets. **(C, D)** Scatter plots showing the causal relationships among the seven negative plasma metabolites and sepsis based on the ieu-b-4981 **(C)** and ieu-b-5086 **(D)** datasets.

Analysis of the causal relationship between metabolite levels and sepsis. 57 plasma metabolites were found causally associated with sepsis (IVW, p < 0.05) of the GWAS ieu-b-4981 dataset. A volcano plot is shown in **Figure 4A**, and detailed data are presented in **Supplementary Table S3**. We identified 63 plasma metabolites with causal relationships with sepsis based on the IVW analysis (p < 0.05) of the GWAS ieu-b-5086 dataset. A volcano plot is shown in **Figure 4B**, and detailed data are presented in **Supplementary Table S4**. The intersection were 13 plasma metabolites which were causal relationship with sepsis, there were bilirubin degradation product,C17H20N2O5 (1) levels, Glutamine degradant levels, Inosine levels, Hydroxypalmitoyl sphingomyelin (d18:1/16:0(OH)) levels, 1-stearoyl-2-arachidonoyl-gpc (18:0/20:4) levels, Docosahexaenoylcarnitine (C22:6) levels, 1-linoleoyl-2-arachidonoyl-GPC (18:2/20:4n6) levels, phosphate to asparagine ratio, Methyl-4-hydroxybenzoate sulfate levels, Malate levels, X-23587 levels, 1-(1-enyl-palmitoyl)-2-oleoyl-gpc (p-16:0/18:1) levels, and 9,10-DiHOME levels respectively, the Venn diagram was presented in **Figure 4C**. Forest plots revealed bilirubin degradation product,C17H20N2O5 (1) levels, Glutamine degradant levels, Inosine levels, X-23587 levels and 9,10-DiHOME levels could increase the risk of sepsis(b>0), 1-(1-enyl-palmitoyl)-2-oleoyl-gpc (p-16:0/18:1) levels, Hydroxypalmitoyl sphingomyelin (d18:1/16:0(OH)) levels, 1-linoleoyl-2-arachidonoyl-GPC (18:2/20:4n6) levels, Docosahexaenoylcarnitine (C22:6) levels, phosphate to asparagine ratio, 1-stearoyl-2-arachidonoyl-gpc (18:0/20:4) levels, Methyl-4-hydroxybenzoate sulfate levels and Malate levels could reduce the risk of sepsis respectively (b<0), the visualization results were displayed in **Figures 5A, B**, **Figures 6A, B**. The casual relation between plasma metabolites and sepsis also were calculated via five methods, and scatter plots exhibited that the greater of SNP effect on 1-(1-enyl-palmitoyl)-2-oleoyl-gpc (p-16:0/18:1) levels, Docosahexaenoylcarnitine (C22:6) levels, 1-linoleoyl-2-arachidonoyl-GPC (18:2/20:4n6) levels, phosphate to asparagine ratio, Methyl-4-hydroxybenzoate sulfate levels, Malate levels, and 1-stearoyl-2-arachidonoyl-gpc (18:0/20:4) levels were associated with a lower sepsis risk, the result shown in the **Figures 5C, D**, the greater of SNP effect on bilirubin degradation product,C17H20N2O5 (1) levels, Glutamine degradant levels, Inosine levels, Hydroxypalmitoyl sphingomyelin (d18:1/16:0(OH)) levels, X-23587 levels and 9,10-DiHOME levels were associated with a higher sepsis risk, the result shown in the **Figures 6C, D**. The data were assessed for bias, and their robustness was confirmed through leave-one-out sensitivity analyses. The plots are provided in **Supplementary Figures S2**, **S3**.

### 3.3 Glutamine degradation mediates SSC-A on HLA DR+ NK association with sepsis

**Figure 7:**
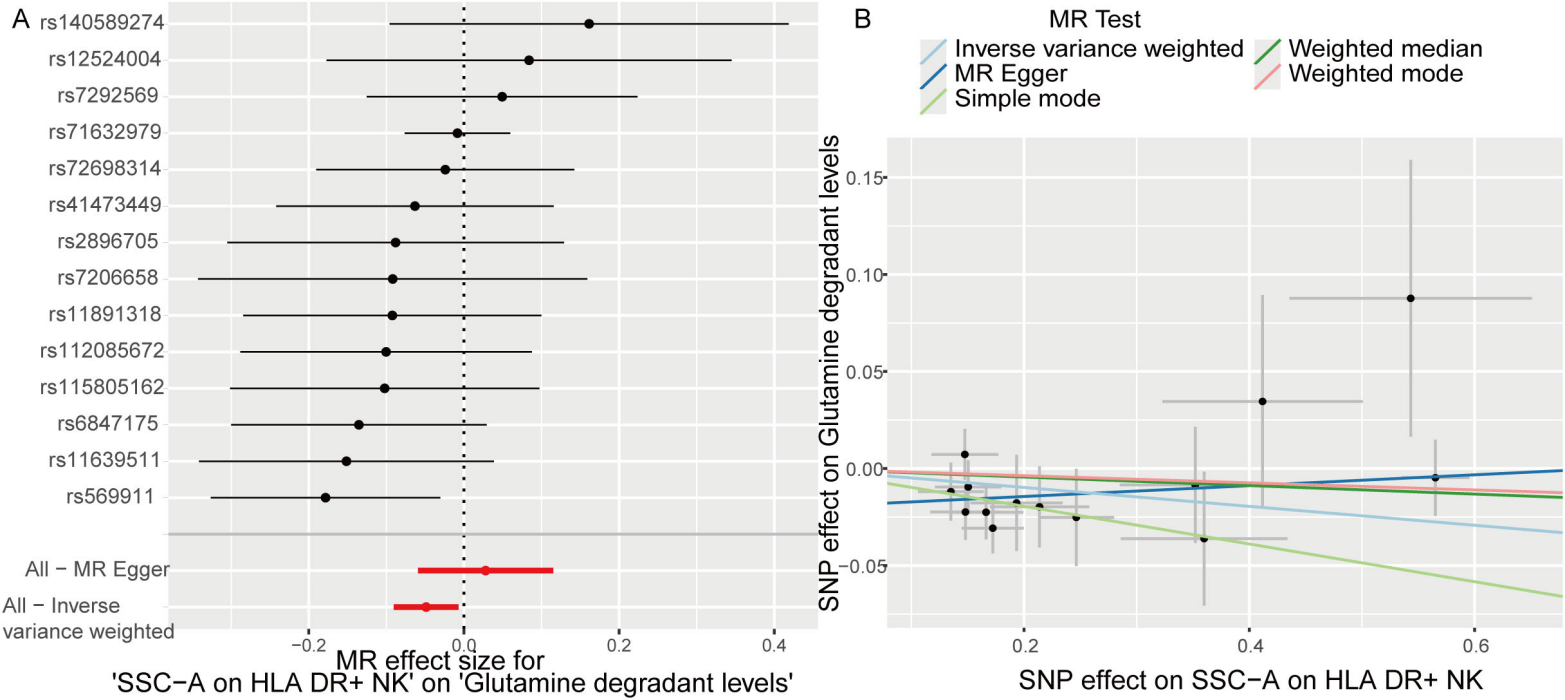
Glutamine degradant levels as a mediator of the relationship between SSC-A on HLA DR+ NK and sepsis. **(A)** Forest plot shown the SSC-A on HLA DR+ NK reduce the risk of sepsis via reducing the level of glutamine degradant(b<0); **(B)** Scatter plots exhibited the greater of SNP effect on SSC-A on HLA DR+ NK, the lower SNP effect on glutamine degradant levels.

TSMR analysis revealed glutamine degradant levels as a mediator linking SSC-A on HLA DR+ NK to sepsis (IVW, p=0.02 and b<0). The result of TSMR analysis was present in the **Table 2**, which was same as the causal relationship between immune phenotype, plasma metabolites and sepsis. The Forest plot identified that the SSC-A on HLA DR+ NK reduce the risk of sepsis via reducing the level of glutamine degradant(b<0), the visualization results were displayed in **Figure 7A**, the casual relation between SSC-A on HLA DR+ NK and glutamine degradant levels also were calculated via five methods and scatter plots exhibited that the greater of SNP effect on SSC-A on HLA DR+ NK, the lower SNP effect on glutamine degradant levels, the diagram was displayed in **Figure 7B**. MR pleiotropic analysis found no pleiotropic effects between SSC-A on HLA DR+ NK and glutamine degradant levels (P > 0.05). The data were subjected to stringent bias evaluations and leave-one-out sensitivity analyses to ascertain the robustness of the MR outcomes. The plots are provided in **Supplementary Figure S3**.

**Table 2:** The mediation MR analysis results of SSC-A on HLA DR+ NK and Glutamine degradant levels.

Outcome	Exposure	Method	nsnp	b	se	pval
Glutamine degradant levels	SSC-A on HLA DR+ NK	MR Egger	14	0.03	0.04	0.54
Glutamine degradant levels	SSC-A on HLA DR+ NK	Weighted median	14	-0.02	0.03	0.45
Glutamine degradant levels	SSC-A on HLA DR+ NK	Inverse variance weighted	14	-0.05	0.02	0.02
Glutamine degradant levels	SSC-A on HLA DR+ NK	Simple mode	14	-0.10	0.06	0.14
Glutamine degradant levels	SSC-A on HLA DR+ NK	Weighted mode	14	-0.02	0.03	0.59

MR, Mendelian randomization; nSNP, non-synonymous single nucleotide polymorphism; pval, p- value.

### 3.4 ScRNA-seq validation of mediation mendelian randomization findings

**Figure 8:**
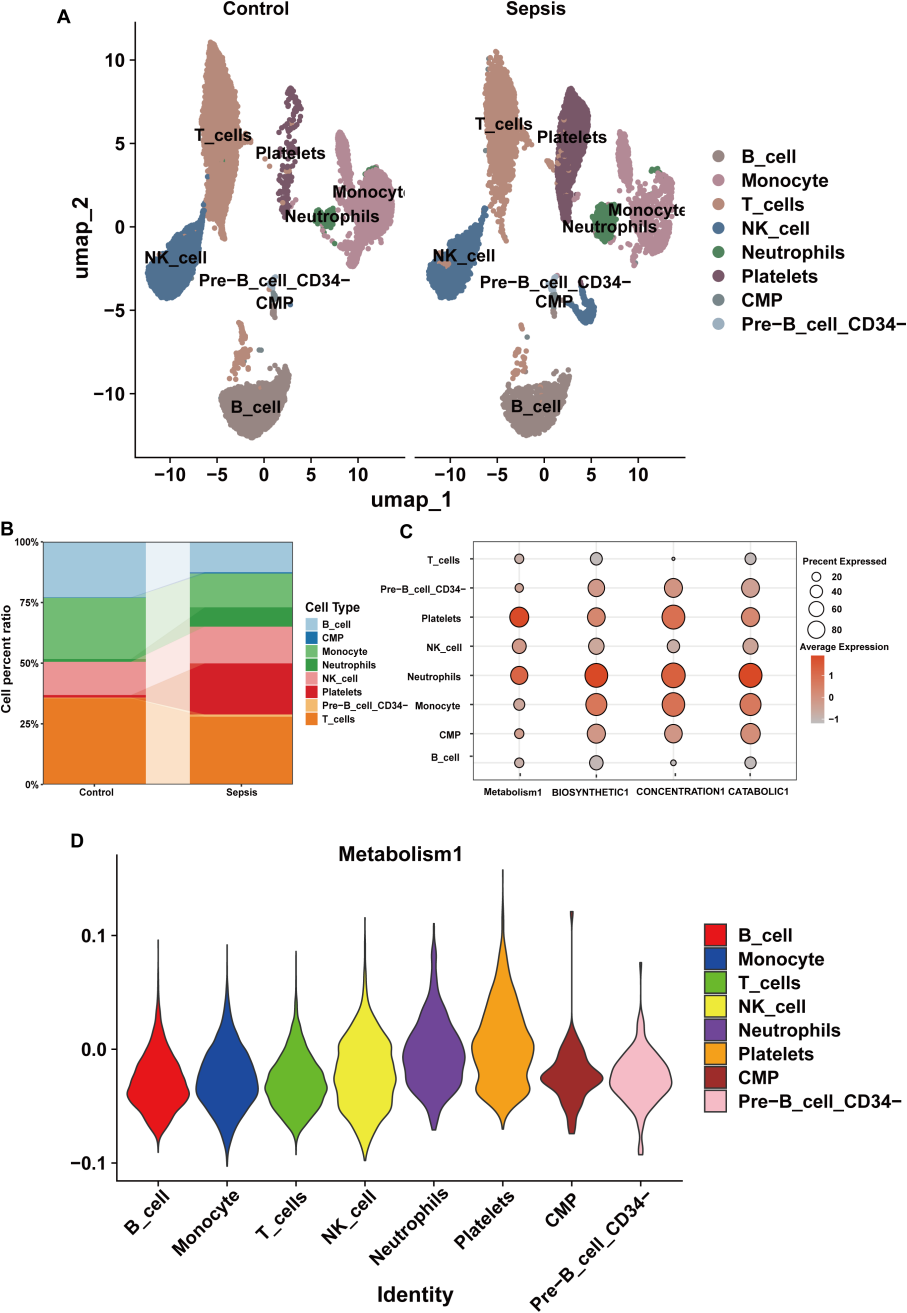
The composition of immune cells and their correlation with glutamine metabolism in the scRNA-seq datasets. **(A)** Annotation of the scRNA-seq datasets using UMAP. **(B)** Immune cell percentage ratios in the control and sepsis groups. **(C)** Bubble chart of the module scores of immune cells for glutamine metabolism and biosynthetic, concentration, and catabolic GSEA pathways. **(D)** Violin diagram of the glutamine metabolic pathway scores for the immune cells.

The cell percentage ratio diagrams and Uniform Manifold Approximation and Projection (UMAP) revealed that the proportions of CMPs, neutrophils, NK cells, platelets, and Pre-B_cell_CD34^-^ cells were higher for the patients with sepsis than for the controls. However, the proportions of B cells, monocytes, and T cells were lower for the patients with sepsis than for the controls (**Figures 8A, B**). We identified that the SSC-A on HLA DR+ NK reduce the risk of sepsis via reducing the level of glutamine degradant based on mediation analysis, SSC-A on HLA DR+ NK reduced the level of glutamine degradant might via decreasing glutamine metabolism. The bubble chart represents the module score of the immune cells for glutamine metabolism, biosynthesis, concentration, and catabolic GSEA pathways. NK cells are definitely related to glutamine metabolism, and in the assessment of glutamine metabolic pathway scores, the violin diagram showed that NK cells occupied the third position, providing additional empirical support for the conclusions derived from the mediation analysis, as shown in **Figures 8C, D**.

**Figure 9:**
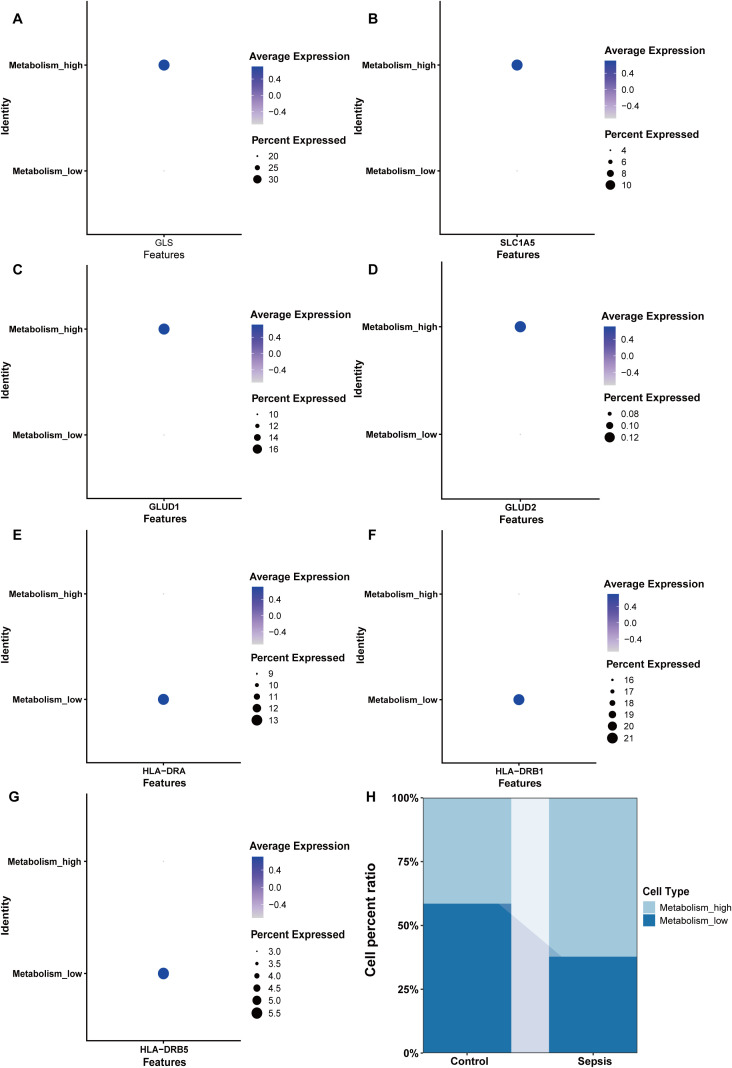
Key enzymes involved in glutamine metabolism and HLA expression in the Metabolism_high and Metabolism_low groups. **(A)** The expression of GLS. **(B)** The expression of SLC1A5. **(C)** The expression of GLUD1. **(D)** The expression of GLUD2. **(E)** The expression of HLA-DRA. **(F)** The expression of HLA-DRB1. **(G)** The expression of HLA-DRB5. **(H)** The proportions of Metabolism_high and Metabolism_low NK cells in the control and sepsis groups. Metabolism_high: NK cell with high glutamine metabolism; Metabolism_low: NK cell with low glutamine metabolism.

NK cells were grouped into high glutamine metabolism groups (metabolism_high) and low glutamine metabolic groups (metabolism_low) based on the glutamine metabolic index. The expression levels of glutaminase (GLS), Solute Carrier Family 1 member 5 (SLC1A5), Glutamate Dehydrogenase 1 (GLUD1), and Glutamate Dehydrogenase 2 (GLUD2) were higher in the metabolism_high group (**Figures 9A-D**). Moreover, we found that HLA-DR + NK cells belonged to the metabolism_low group, and the results for HLA-DRA, HLA-DRB1, and HLA-DRB5 expression are shown in **Figures 9E-G**. The sepsis group had more metabolism-high NK cells and fewer metabolism-low NK cells than the control group. This indicated increased glutamine degradation in sepsis, which was consistent with the findings of our MR analyses (**Figure 9H**).

### 3.5 GSEA analysis

**Figure 10:**
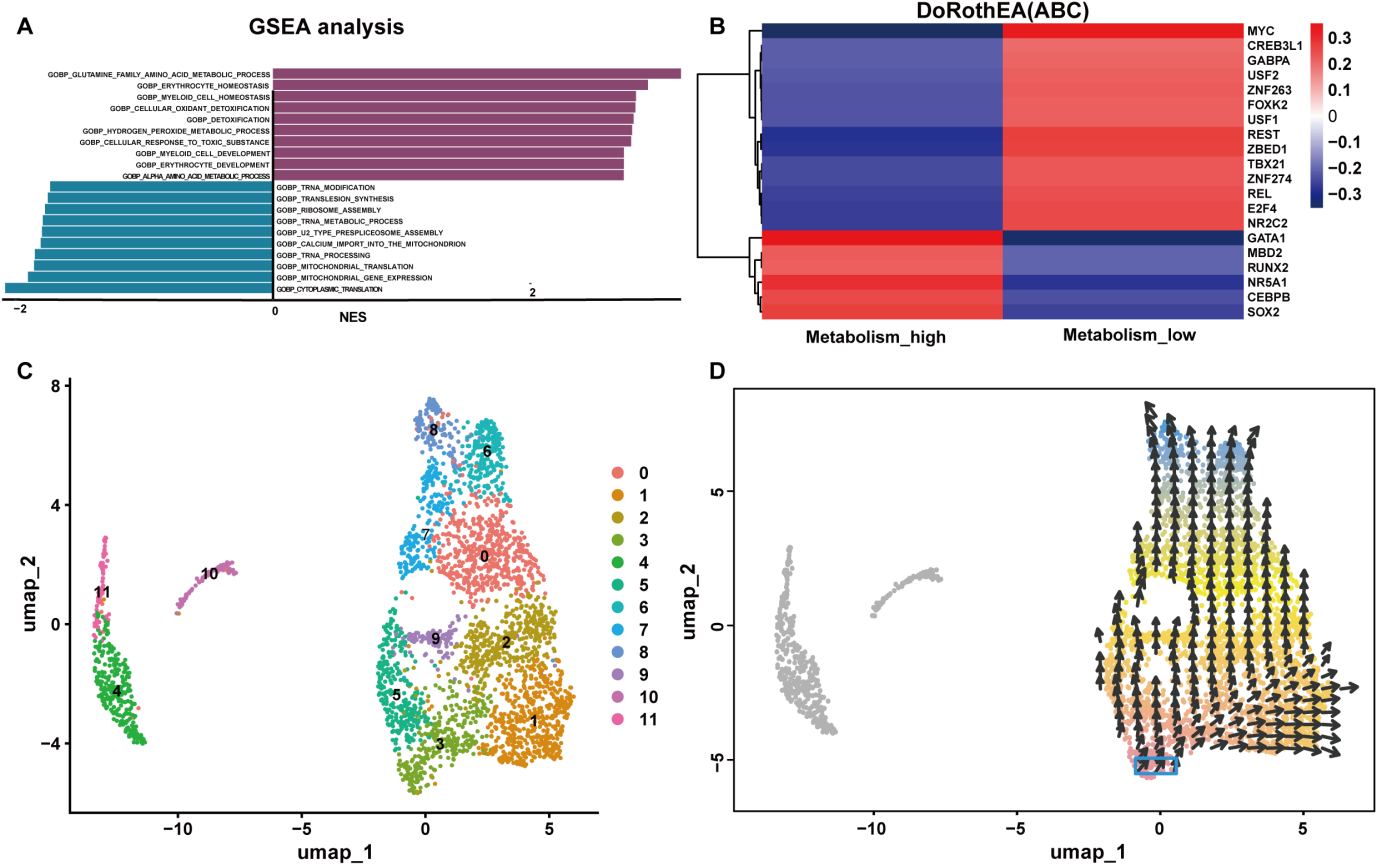
Functions of NK cells with high and low glutamine metabolism. **(A)** GSEA of the differentially expressed genes in the metabolism_high and metabolism_low groups. **(B)** The transcription factor prediction for the metabolism_high and metabolism_low groups. **(C, D)** The cellular pseudotime plots of the NK cells. NES, Normalized Enrichment Score.

The “FindMarkers” function was used to identify 9955 DEGs in the metabolism_high and metabolism_low groups. GSEA analysis illustrated the positively correlated with differential genes were GOBP GLUTAMINE FAMILY AMINO ACID METABOLIC PROCESS, GOBP ERYTHROCYTE HOMEOSTASIS and GOBP MYELOID CELL HOMEOSTASIS as the top three pathways positively associated with these genes. The top 3 pathways negatively associated with these genes were GOBP TRNA MODIFICATION, GOBP TRANSLESION SYNTHESIS, GOBP RIBOSOME ASSEMBLY. These findings are illustrated in the column chart in **Figure 10A**.

### 3.6 Transcription factor activity prediction and cellular pseudotime analysis

TF prediction analysis indicated that the metabolism_high NK cells and metabolism_low NK cells groups were characterized by opposite TF profiles. For instance, metabolism_high NK cells were positively correlated with GATA Binding Protein 1(GATA1) and nuclear receptor subfamily 5 group A member 1 (NR5A1), and metabolism_high NK cells showed a significant inverse correlation with myelocytomatosis oncogene (MYC), RE1 silencing transcription factor (REST), and zinc finger BED-type containing 1(ZBED1). Consequently, there were positive correlations between metabolism_low NK cells and MYC, REST, and ZBED1, and negative correlations between metabolism_low NK cells and GATA1 and NR5A1. A hot map of the transcription factor prediction is shown in **Figure 10B**. The cellular pseudotime plot is illustrated in **Figures 10C, D**, which delineates the ontogenetic progression of NK cells and depicts their developmental trajectory from their inception to full maturity.

### 3.7 Development of sepsis prognostic models via machine learning

**Figure 11:**
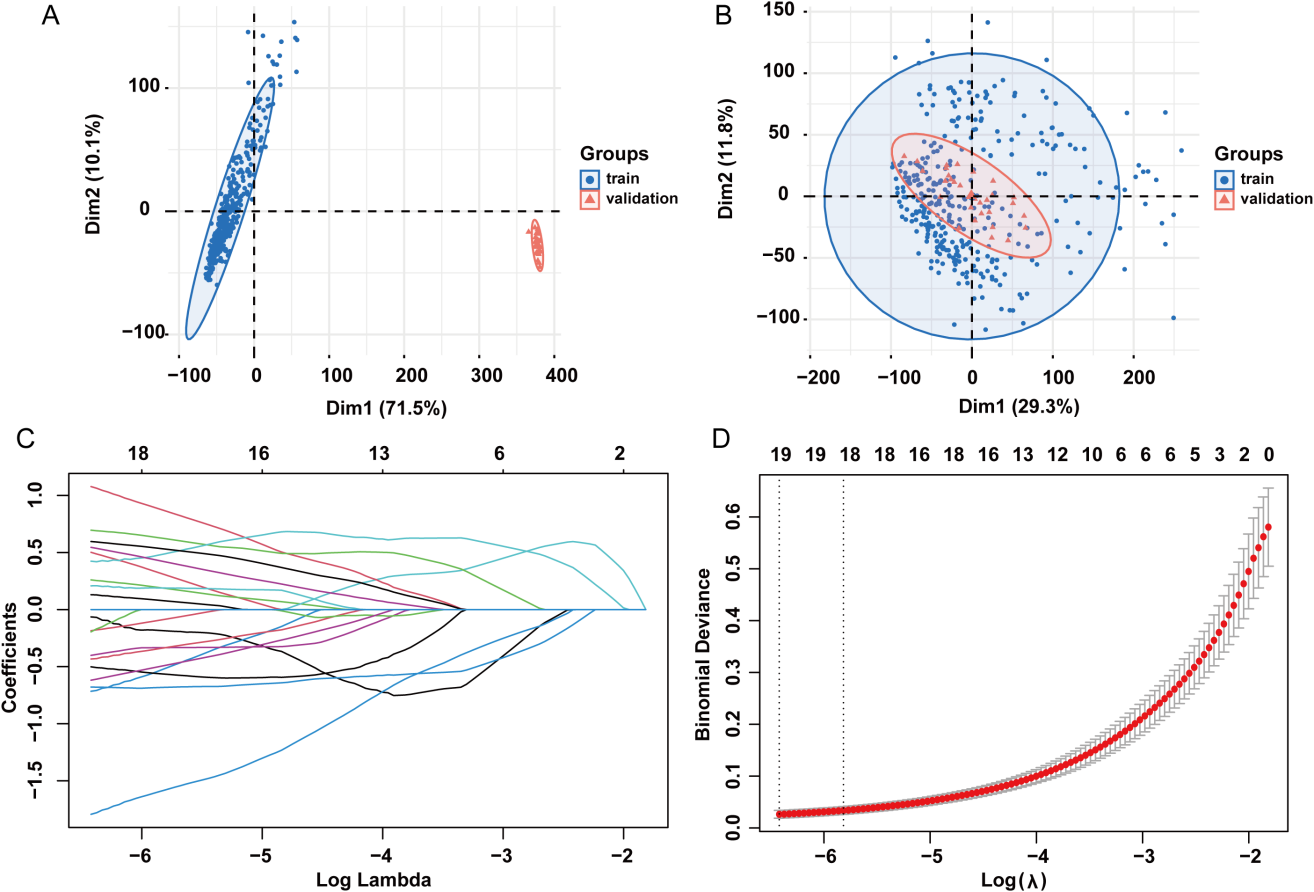
LASSO regression analysis. **(A)** PCA plots showing variance in the dataset before batch effect correction. **(B)** PCA plots showing variance in the dataset after batch effect correction. **(C)** LASSO coefficient path plot. **(D)** Cross-validation curve for lasso analysis. LASSO: least absolute shrinkage and selection operator.

**Figure 12:**
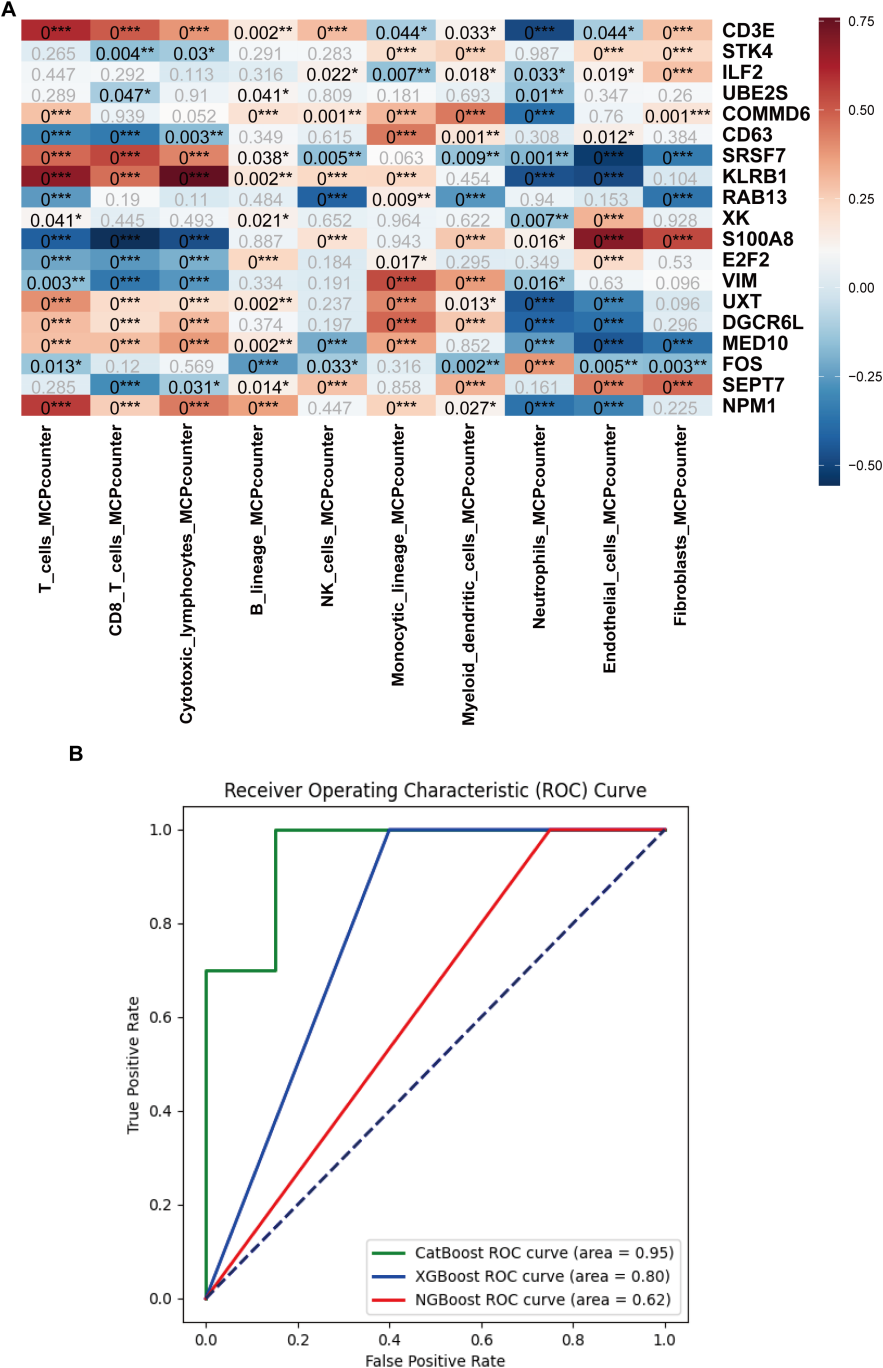
Immune cell infiltration analysis and machine learning model development based on the hub genes. **(A)** Heatmap of the immune cell infiltration analysis. **(B)** AUC curves of the CatBoost, XGBoost, and NGBoost ML models for the test set. The AUCs of CatBoost, NGBoost, and XGBoost were 0.95, 0.62, and 0.80, respectively (*:p ≤ 0.05; **:p ≤ 0.01; ***:p ≤ 0.001).

Bulk datasets GSE236713 and GSE28750 successfully removed batch effects, and the PCA plots depicted the variance in the dataset before and after batch-effect correction, as illustrated in **Figures 11A, B**. 789 genes were obtained via the intersection analysis between 9955 DEGs and the DEGs in the training bulk dataset GSE236713. LASSO regression analysis found 19 pivotal hub genes (**Figures 11C, D**). The immune infiltration heatmap demonstrated most hub genes are positively correlated with the regulation of T cell, CD8 T cell, cytotoxic lymphocyte, and monocyte lineage MCP counter, and most hub genes are negative correlation with the regulation of neutrophil, endothelial cell, and fibroblast MCP counter (**Figure 12A**).

**Figure 13:**
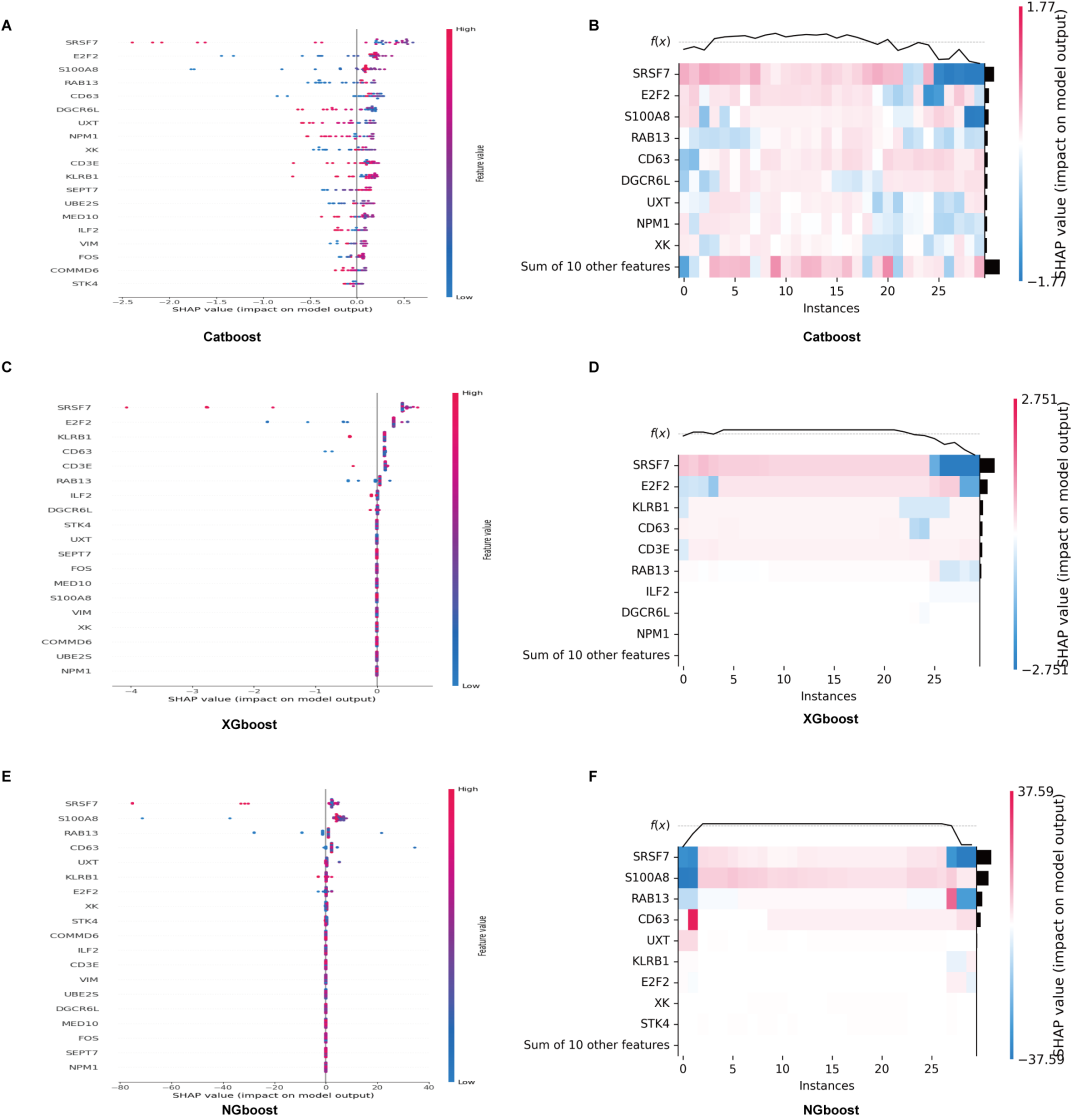
SHAP analysis of the significance of CatBoost, XGBoost and NGBoost. **(A, B)** Beeswarm and heatmap plots show that the top three important genes for XGBoost are SRSF7, E2F2, and KLRB1. **(C, D)** Beeswarm and heatmap plots show that the top three important genes for CatBoost are SRSF7, E2F2, and S100A8. **(E, F)** Beeswarm and heatmap plots show that the top three important genes for NGBoost are SRSF7, S100A8, and RAB13.

Three boosting algorithms were employed to analyze 19 genes and construct predictive models. In test set, their Area Under the Curve (AUC) of CatBoost ML algorithms was 0.95, XGboost was 0.80, NGboost was 0.62, the AUC values for all three algorithms were greater than 0.6, confirmed the model had a high diagnostic efficacy, the Receiver Operating Characteristic Curve chart was displayed in **Figure 12B**. SHAP was used to analyze the significance of the three ML algorithms, and the Beeswarm Plots and Heatmap Plots showed that the top three important genes were SRSF7, E2F2, and KLRB1 for XGBoost; SRSF7, E2F2, and S100A8 for CatBoost; and SRSF7, S100A8, and RAB13 for NGBoost (**Figure 13**).

### 3.8 Identified the expression of significant genes in sepsis

**Figure 14:**
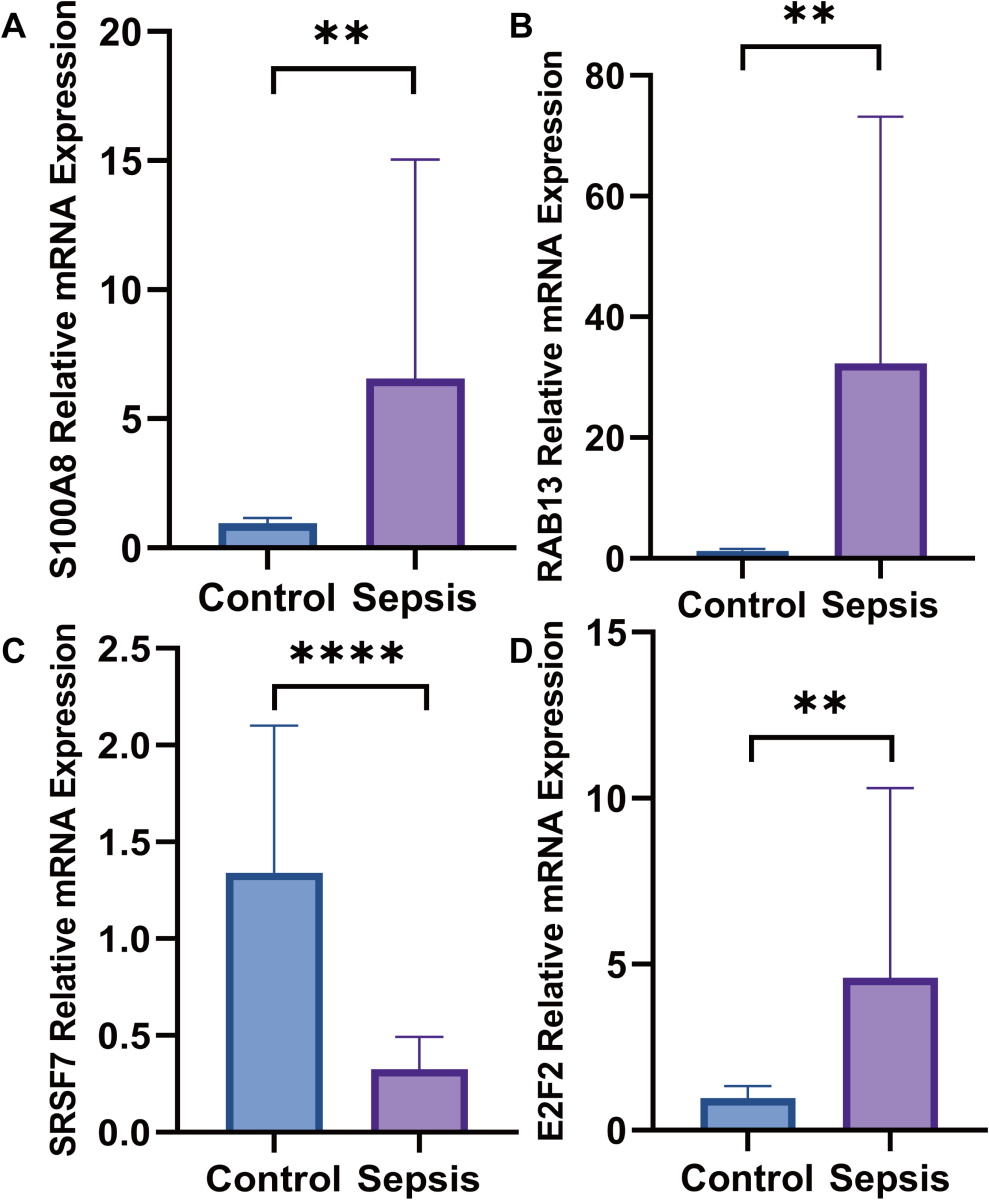
The mRNA expression levels. **(A)** The mRNA expression level of S100A8 was higher in septic patients than healthy people; **(B)** The mRNA expression level of RAB13 was higher in septic patients than healthy people; **(C)** The mRNA expression level of SRSF7 weas lower in septic patients than healthy people; **(D)** The mRNA expression level of E2F2 was higher in septic patients than healthy people (**:p ≤ 0.01; ****:p ≤ 0.0001).

The PBMC were separated of twenty patients with sepsis and twenty healthy volunteers, we estimated the mRNA expression level of SRSF7, E2F2, RAB13 and S100A8 in PBMC via RT-qPCR, the mRNA expression level of SRSF7(p<0.0001) was lower in the patients with sepsis than healthy people. In contrast, the expression level of E2F2(p=0.0073), RAB13(p=0.0016) and S100A8(p=0.0053) were higher, the results were shown in **Figure 14**.

## 4 Discussion

Sepsis is associated with a significant disease burden and considerable morbidity and health care costs. It accounts for approximately 20% of all deaths globally (41). However, the exact mechanisms underlying its pathogenesis have not been elucidated. Empirical studies have suggested that the pathogenesis of sepsis is intricately linked to the dysregulation of both immune and metabolic processes. However, the underlying mechanisms are not yet completely understood (42, 43). In our study, we utilized mediation analysis, scRNA-seq analysis and ML algorithms to identify glutamine degradant levels as a mediator linking SSC-A on HLA DR+ NK to sepsis. As a protective factor, SSC-A on HLA-DR + NK cells were reduced the risk of sepsis, whereas glutamine degradant levels were increased the risk of sepsis. Consequently, in our scRNA-seq analysis, glutamine metabolic activity in HLA-DR+ NK cells was reduced and HLA-DR+ NK cells exhibited reduced glutamine metabolic activity. The number of NK cells with low glutamine metabolism decreases, whereas that of NK cells with high glutamine metabolism increases in sepsis. This metabolic shift within the NK cell subset is associated with the elevated production of glutamine degradant levels, thereby augmenting the risk of sepsis. Finally, we constructed a model and explored 19 hub genes that may be useful for early diagnosis and treatment of sepsis.

Immunity take part in the pathogenesis and progression of sepsis (44). Most scholars attribute the high mortality rate of sepsis to multiorgan failure secondary to immunological damage caused by excessive inflammatory responses (1, 45). This is specifically manifested by the concurrent dysregulation of the innate immune system and suppression of adaptive immunity (46). Given the integral involvement of immunity in the pathogenesis of numerous diseases, therapeutic interventions targeting immune pathways represent a promising strategy. Research demonstrates that targeted elimination of senescent cells within the immune microenvironment can impede tumor progression and metastasis (47). To identify novel immunotherapeutic targets for sepsis, comprehensive investigation into causal relationships between immune and sepsis is essential. In our study, MR analysis showed that IgD+ CD24+ %B cells, CD45 on CD8br, SSC-A on HLA-DR + NK, and CD8br AC were reduced risk of sepsis, whereas SSC-A on plasmacytoid DC was increased risk of sepsis. CD45 on CD8br participate in immune-related diseases too. Studies have shown that the amount of CD45 on CD8br is lower in Primary Sjögren’s syndrome (pSS) than in healthy individuals, and CD45 on CD8br may be related to a reduced risk of pSS (48). CD8br AC is an essential immune cell phenotype that has been reported to have a causal relationship with lymphocytic leukemia, epilepsy, and autism spectrum disorders (49–51). IgD+CD24+B cells belong to subpopulation of B cells, and their phenotypes and functions are important in immunological research. IgD+CD24+B cells are associated with osteoporosis and may reduce its risk (52). SSC-A on HLA-DR + NK cells is a subpopulation of NK cells characterized by HLA-DR surface expression levels. SSC-A reflects both size and complexity. Studies reported that HLA-DR is a biomarker of NK cell activation and proliferation (53). HLA-DR + NK cells is correlates with the inflammation level in the peripheral blood, and if overactivated, its own tolerance can be destroyed (54–56), in addition, HLA-DR + NK cells are strongly related with reduced tumors risk (57). SSC-A is a phenotype related to lysosomal function and secretion and can be distinguished using flow cytometry technology (58, 59). At present, there is little research on SSC-A on HLA-DR + NK cells and its function is dual. For instance, SSC-A on HLA-DR + NK cells increased risk of bone and cartilage cancers (60). In our study, SSC-A on HLA-DR + NK cells was a protective factor and associated with a reduced risk of sepsis. SSC-A on plasmacytoid DC also has dual effects on various diseases. Zhao et al. reported that SSC-A on plasmacytoid DC might have a causal relationship with an increased risk of epilepsy (50).

Metabolic dysregulation is pivotal to the pathogenesis and progression of sepsis (61). For instance, metabolic dysregulation of lipids and cholesterol can cause or aggravate sepsis by regulating inflammation level (62). In our study, we found that the bilirubin degradation product, C17H20N2O5 (1), glutamine degradant, and inosine levels were associated with increased risk of sepsis. Conversely, 1-linoleoyl-2-arachidonoyl-GPC (18:2/20:4n6), docosahexaenoylcarnitine (C22:6), phosphate to asparagine ratio, 1-(1-enyl-palmitoyl)-2-oleoyl-gpc (p-16:0/18:1) levels 5086, Methyl-4-hydroxybenzoate sulfate levels, hydroxypalmitoyl sphingomyelin (d18:1/16:0(OH)), malate, X-23587, 1-stearoyl-2-arachidonoyl-gpc (18:0/20:4), and 9,10-DiHOME levels were associated with a reduced risk of sepsis. For example, bilirubin degradation product is related to the inflammatory response (63), inosine is associated with sepsis caused by viruses (64) and malate is a potential biomarker for septic shock (65). Glutamine degradant levels are metabolites with a significant causal relationship with sepsis. Glutamine degradants include all metabolic products of glutamine such as glutamic acid, carbon dioxide, aspartic acid, and so on. In the human, glutamine as a non-essential amino acid is closely associated with nitrogen transport, proliferation, immune cell function, maintenance of the intestinal barrier function, and muscle recovery (66). Karinch reported a significant metabolic disorder involving glutamine concentrations in various organs and tissues. The intestinal absorption of glutamine decreases during sepsis, but its metabolism in the liver and immune cells increases. This imbalance contributes to the compromise of the intestinal barrier, increased production of inflammatory mediators, and exacerbation of sepsis (67). In our study, we identified glutamine degradant level as a potential risk factor for sepsis, which was causally related to an increased risk of sepsis. Therefore, we speculated that increased glutamine metabolism might lead to higher levels of glutamine degradants, thereby increasing the risk of sepsis.

Mediation MR analysis identified specific mechanisms and connections between the three immune cell phenotypes, metabolism, and sepsis. We found that the glutamine degradant level as a mediator in the causal relationship between SSC-A on HLA-DR + NK cells and sepsis, and SSC-A on HLA-DR + NK cells reduced the risk of sepsis by diminishing the glutamine degradant level.

MR results were further substantiated by sc-RNA seq analysis. First, we explored the differences in the compositions of immune cells in the sepsis and control groups. The adaptive immune cells, especially B and T cells, were less abundant in the sepsis group, which was consistent with the pathogenesis of sepsis. Many researches found CD4+ and CD8+T cells are diminished (13, 68) and that the amount, phenotype, and function of B cells are changed in sepsis (69). We observed increased neutrophil and platelet counts in septic patients. Extensive studies indicate that circulating neutrophils are elevated during sepsis, predominantly in an immature state (70). Neutrophils exhibit dual functionality: they initiate antimicrobial activities through secretion of proteolytic enzymes and reactive oxygen species, yet uncontrolled neutrophil activation may drive pro-inflammatory responses associated with multi-organ injury (71). Elevated S100A8 levels may also originate from neutrophil mobilization (72). Regarding glutamine interactions, studies demonstrate that glutamine reduces neutrophil tissue infiltration and mitigates inflammatory responses in septic mice (73).While platelets are established mediators of hemostasis and thrombosis, their immunomodulatory roles are increasingly recognized (74). Platelets contribute to immunity through inflammatory mediator release, immune molecule expression, and cross-talk with immune cells (75). In sepsis, splenic-derived protective platelet populations express high levels of CD40 ligand and release inflammatory mediators to engage in immune defense (76). As sepsis progresses, platelet proportions significantly increase while platelet-B cell communication markedly declines, underscoring platelet involvement in sepsis-induced immune dysregulation and adverse clinical outcomes (77).Concerning glutamine relationships, research shows glutamine upregulates Platelet-Derived Growth Factor expression during early and late sepsis, modulating local cerebral immune defenses (78). However, the impact of platelets and neutrophils on glutamine metabolism in sepsis remains unclear.

Second, to confirm the relationship between SSC-A on HLA-DR + NK cells and glutamine metabolism, we found HLA-DR + NK cells belonged to the low glutamine metabolism group and were decreased in sepsis. Consequently, the results of MR analysis verified that HLA-DR + NK cells reduce the risk of sepsis by reducing glutamine metabolism and glutamine degradant levels. GSEA was performed to evaluate the functions of the different genes in the metabolism_high and metabolism_low groups. The top pathway positively correlated with the differential genes was the GOBP GLUTAMINE FAMILY AMINO ACID METABOLIC PROCESS, which further validated the grouping. The top three pathways negatively associated with the DEGs were GOBP TRNA MODIFICATION, GOBP TRANSLESION SYNTHESIS, and GOBP RIBOSOME ASSEMBLY. These pathways are all related to RNA and protein synthesis. Several studies have reported the involvement of glutamine metabolism in the carbon metabolism of carbohydrates and proteins and its participation in the synthesis of some cells (79, 80). In addition, we predicted the transcription factors for different genes related to metabolism_high and metabolism_low NK cells and found that the transcription factors were entirely the opposite: metabolism_high NK cells were positively correlated with GATA1 and NR5A1. GATA1 take part in hematopoietic homeostasis and transcription factor regulation, GATA1 could participate in the differentiation of red blood cells and megakaryocytes (81). NR5A1 is closely related to gonadal development and NR5A1 mutations can cause 46, XX DSD and 46, XY DSD (82). Metabolism _high NK cells were significantly negatively correlated with MYC, REST, and ZBED1 expression. Cellular metabolic processes, apoptotic mechanisms, and proliferative activities are intricately linked to MYC transcription factor (83). MYC can reprogram glucose metabolism in erythroleukemia cells and is involved in the occurrence and development of nasopharyngeal carcinoma (84, 85). REST can act as an oncogene and a tumor suppressor. ZBED1 correlates with cell proliferation, with elevated expression promotes cell proliferation and apoptosis in gastric cancer (86). A temporal cellular diagram was created to elucidate the trajectory of NK cell differentiation.

Nineteen hub genes were identified combined multi-method approach. Immune cell infiltration analysis elucidated both positively and negatively correlated cell populations. We then used the 19 hub genes to develop a prediction model based on the CatBoost, XGBoost, and NGBoost ML algorithms. All three methods achieved AUC values exceeding 0.6, CatBoost had the highest AUC (0.95), followed by XGBoost and NGBoost. Research indicates that CatBoost excels at handling categorical features and missing data while mitigating overfitting. These capabilities making it particularly suitable for biomedical and bioinformatics datasets rich in categorical features (87). XGBoost offers comparatively faster computational speed and enhanced model performance (88), whereas NGBoost generates predictive distributions (89). CatBoost, XGBoost, and NGBoost are widely employed in biomedical research for classification and regression due to their flexibility, scalability, and ease of use. Their accuracy and reliability in predicting disease risk and mortality have been validated in multiple studies (90, 91). Compared to algorithms such as LightGBM, Multi-Layer Perceptron, AdaBoost, Logistic Regression, and Support Vector Machines, these three models demonstrate superior classification stability and reliability, effectively reducing overfitting while enhancing the capacity to capture complex data patterns (92). Empirical evidence confirms that CatBoost, XGBoost, or NGBoost models exhibit significant performance advantages in disease risk prediction relative to other ML approaches (93, 94).In summary, given that our data comprise bioinformatics and medical datasets with substantial categorical features, we employed CatBoost, XGBoost, and NGBoost, all of which demonstrated strong predictive value.

SRSF7, E2F2, RAB13 and S100A8 were obtained via SHAP analysis. Their expression levels were determined using RT-qPCR. In sepsis, SRSF7 was lower expression. However, E2F2, RAB13, and S100A8 were higher expression. SRSF7 encodes an serine/arginine (SR)-rich protein involved in pre-mRNA. These proteins are essential components of the spliceosome. SR proteins have been demonstrated to participate in the nuclear export of mRNA and translational regulation, in addition to their critical role in mRNA splicing (95). For example, SRSF7 regulates IRF7 transcription to promote antiviral response in macrophages (96). E2F2 is a member of the E2F family the E2F family can regulate the cell cycle and mediate the functions of tumor suppressor proteins, and E2F2 expression has been identified as a potential diagnostic marker for ARDS secondary to sepsis (97). RAB13 is a member of the RAB small GTPase family within the Ras superfamily. It primarily regulates intracellular vesicle trafficking, cell polarity establishment, and dynamic modulation of cell junctions (98). RAB13 expression is upregulated in sepsis and positively associated with disease severity (99). RAB13 promotes macrophage M1 polarization in sepsis (100). Our study revealed that elevated RAB13 expression associated with increased mortality and incidence of sepsis. S100A8 can inhibit casein kinase and act as a cytokine. Research has reported higher S100A8 expression in patients with sepsis than in controls (101). Elevated S100A8 expression exacerbates sepsis-induced organ dysfunction (102, 103).

Our study elucidated the roles of SSC-A on HLA-DR + NK cells and glutamine metabolism in sepsis using MR and ScRNA-seq analysis. This provides a theoretical foundation for the potential use of SSC-A on HLA-DR + NK cell activity and glutamine metabolite levels as early predictive and diagnostic biomarkers for sepsis. However, our study has certain limitations, despite implementing multiple approaches to minimize false positives, including validation at single-cell level and in clinical cohorts, the absence of multiple testing control during exposure-outcome causality analysis sustains residual false positive risk. Consequently, further verification using clinical samples to validate the relationships and molecular mechanisms are warranted. These will be the focus of our future research.

In summary, our study demonstrated that SSC-A on HLA DR+ NK cells has a protective effect against sepsis by reducing glutamine metabolism and the associated glutamine degradants levels. Finally, we employed multi-omics analysis and ML algorithms to screen for key molecules and develop and validate predictive models with excellent diagnostic performance.

## 5 Conclusion

SSC-A on HLA-DR + NK cells reduced the risk of sepsis by decreasing glutamine degradation. SRSF7, E2F2, RAB13, and S100A8 were identified as potential pathogenic biomarkers of sepsis.

## Data Availability

The original contributions presented in the study are included in the article/**Supplementary Material**. Further inquiries can be directed to the corresponding author.
