# Supplementary material for: Identification of sepsis biomarkers through glutamine metabolism-mediated immune regulation: a comprehensive analysis employing mendelian randomization, multi-omics integration, and machine learning
Source: Front Immunol. 2025 Aug 20;16:1640425. doi: 10.3389/fimmu.2025.1640425 (PMC12404944; doi:10.3389/fimmu.2025.1640425)
Supplement: Supplementary file 1 [file DataSheet1.docx]

Supplementary Material

# Supplementary Figures and Tables

## Supplementary Figures
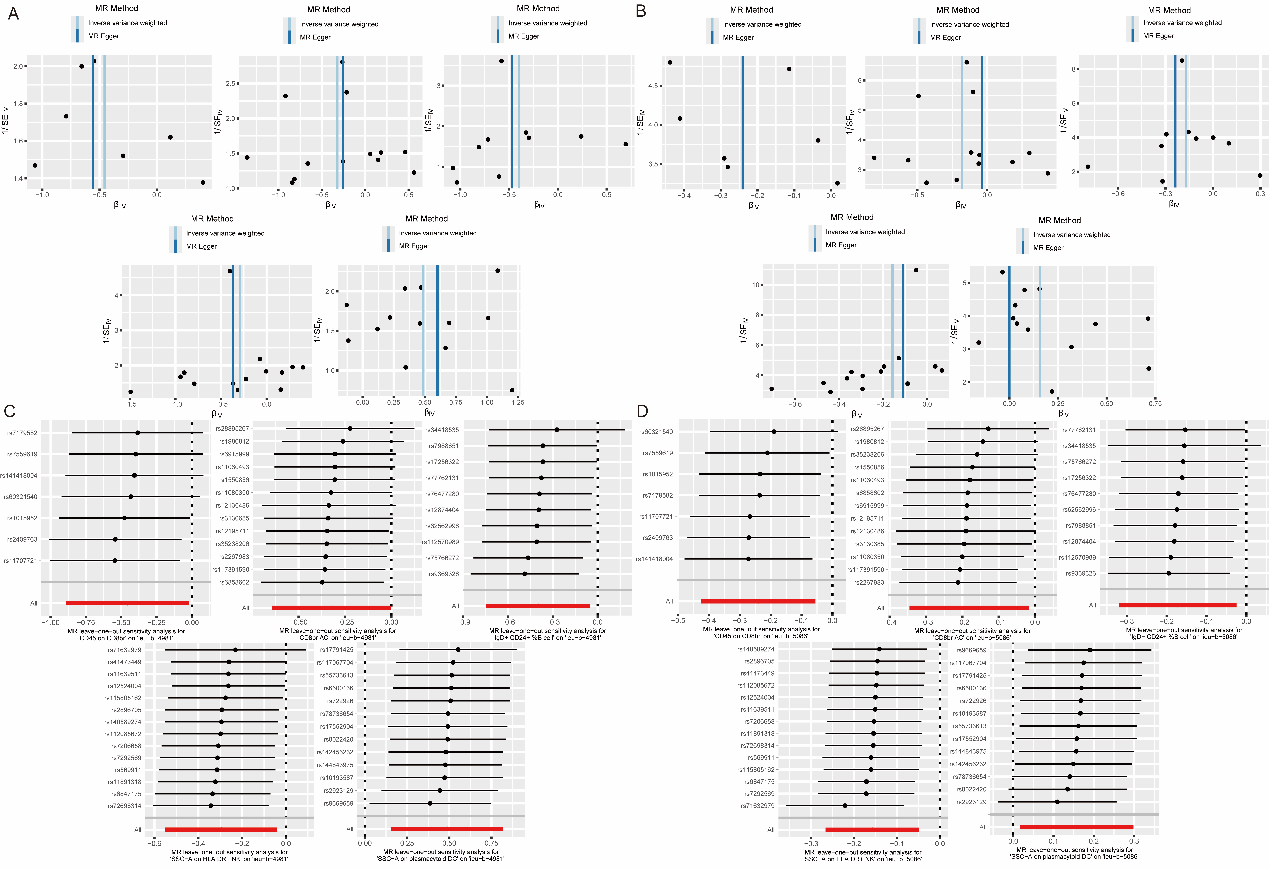


**Supplementary Fig.S1:** Bias assessments and leave-one-out sensitivity analyses in the casual relationship between immune phenotype and sepsis. (A) The funnel plot about bias assessments of the relationship between 5 immune phenotypes and ieu-b-4981. (B) The funnel plot about bias assessments of the relationship between 5 immune phenotypes and ieu-b-5086.
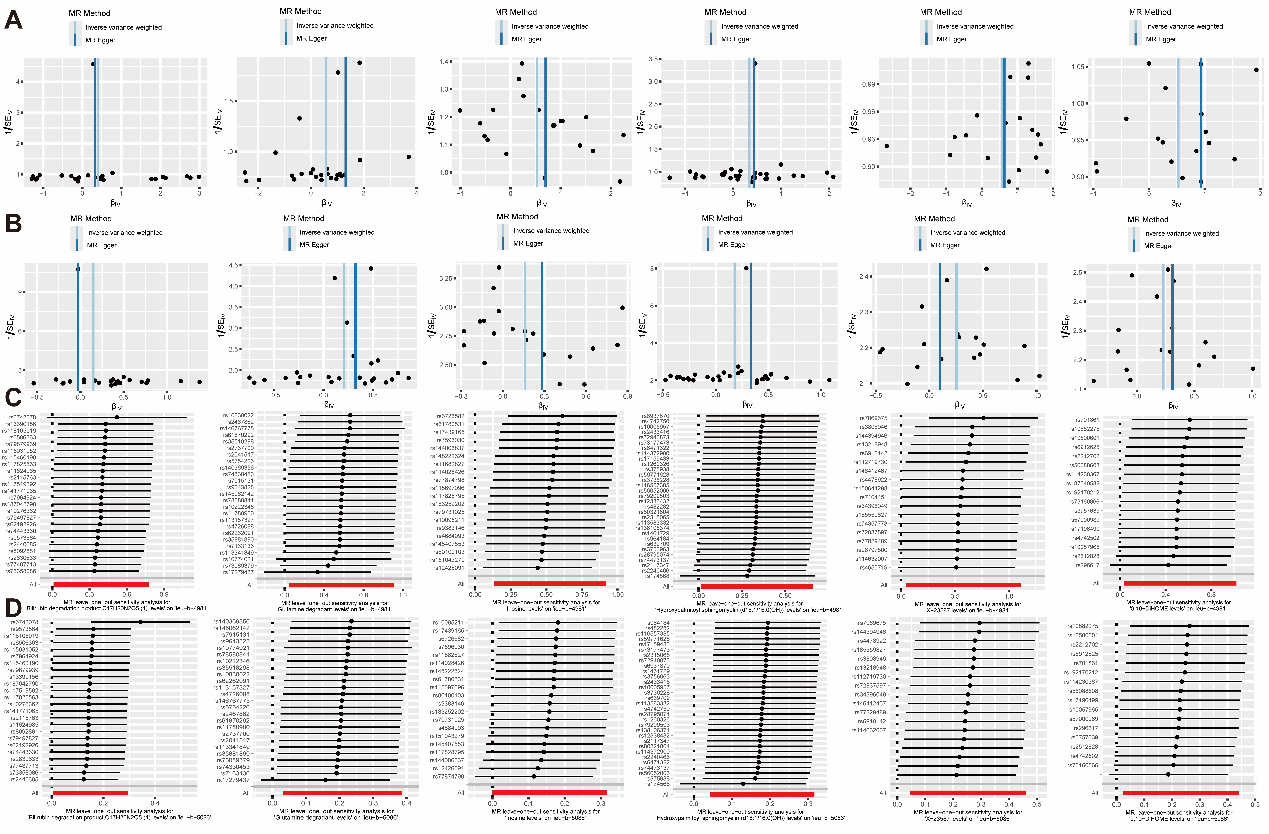
(C) The leave-one-out plot about sensitivity analysis of the association between 5 immune phenotypes and ieu-b-4981. (D) The leave-one-out plot about sensitivity analysis of the correlation b between 5 immune phenotypes and ieu-b-5086.


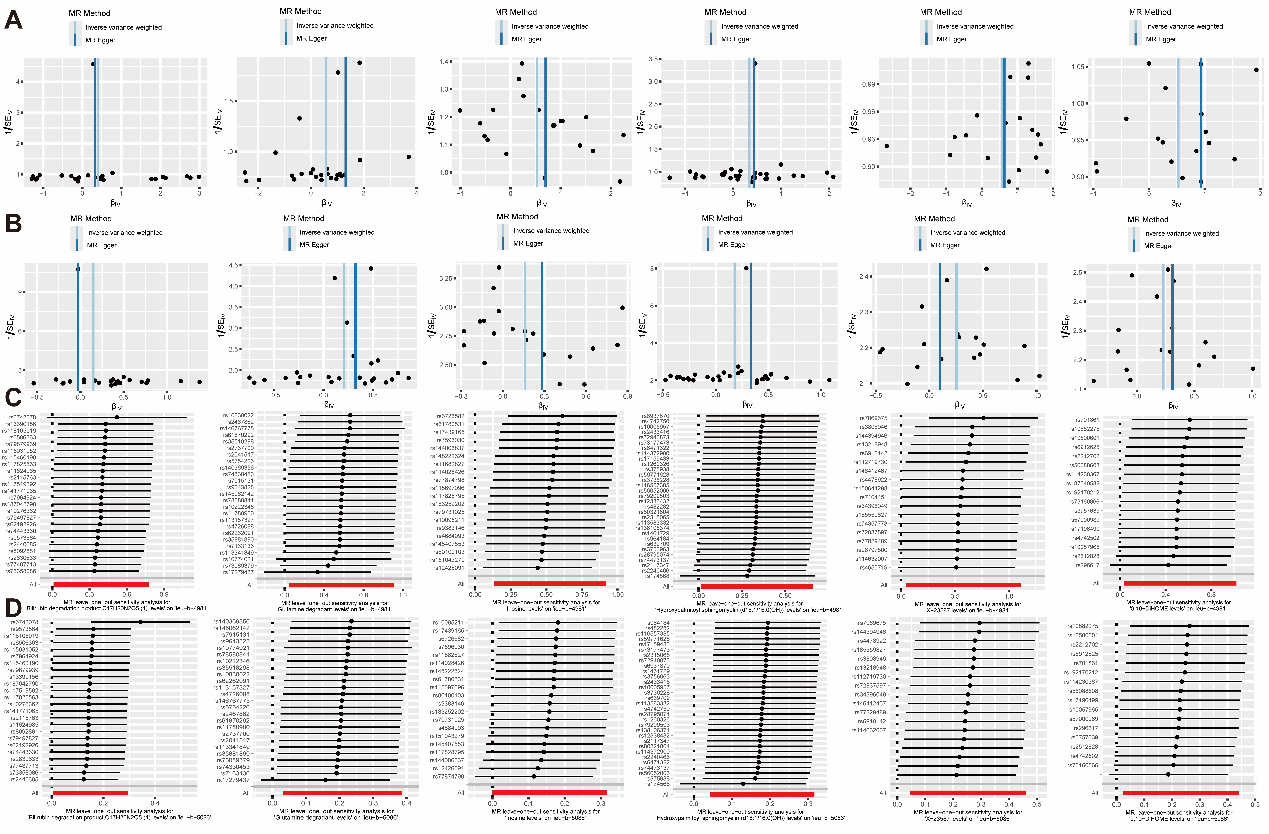


**Supplementary Fig.S2:** Bias assessments and leave-one-out sensitivity analyses in the casual relationship between 6 positive plasma metabolites and sepsis. (A) The funnel plot about bias assessments of the relationship between 6 positive plasma metabolites and ieu-b-4981. (B) The funnel plot about bias assessments of the relationship between 6 positive plasma metabolites and ieu-b-5086. (C) The leave-one-out plot about sensitivity analysis of the association between 6 positive plasma metabolites and ieu-b-4981. (D) The leave-one-out plot about sensitivity analysis of the correlation b between 6 positive plasma metabolites and ieu-b-5086.


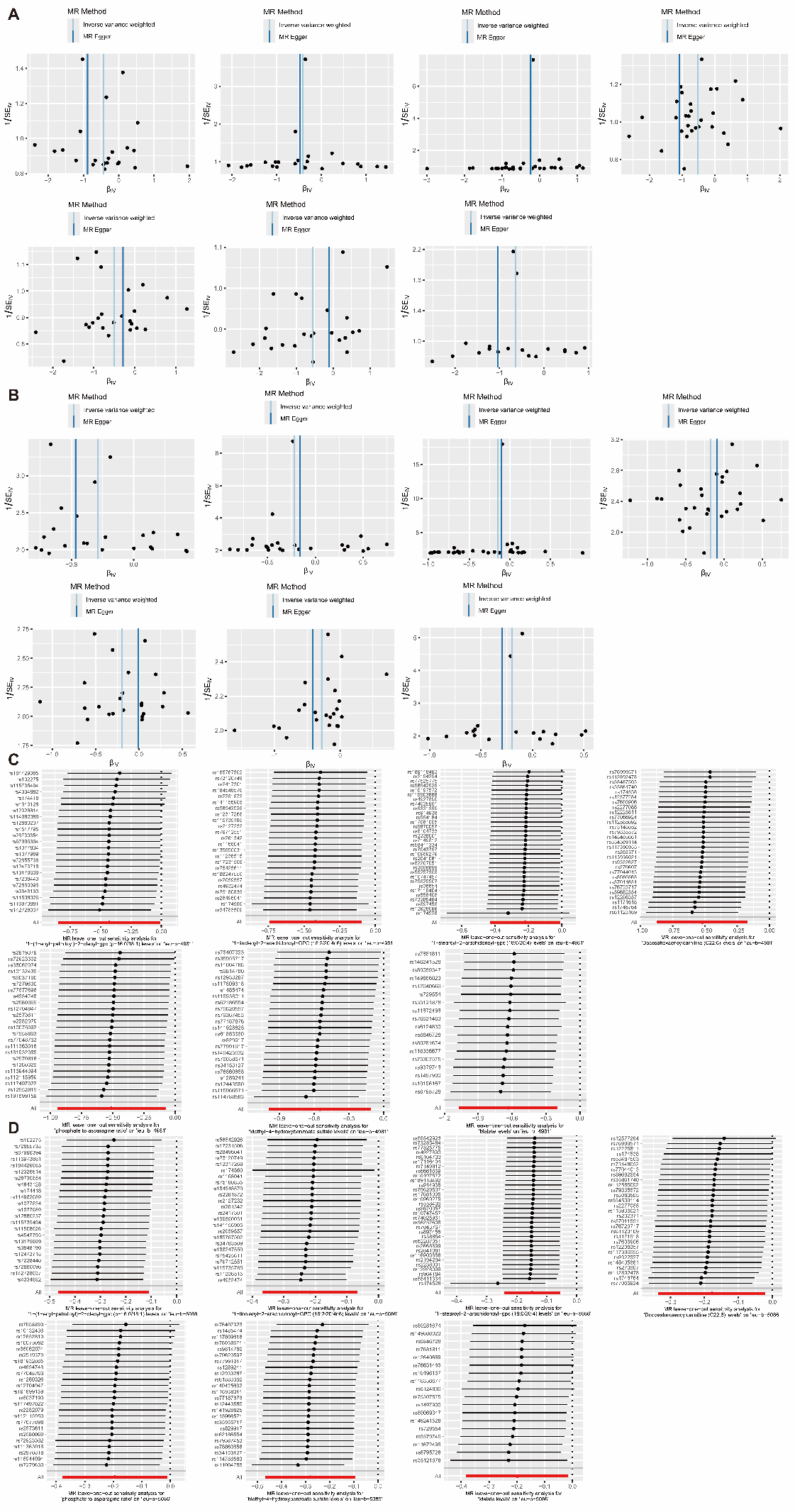


**Supplementary Fig.S3:** Bias assessments and leave-one-out sensitivity analyses in the casual relationship between 7 negative plasma metabolites and sepsis. (A) The funnel plot about bias assessments of the relationship between 7 negative plasma metabolites and ieu-b-4981. (B) The funnel plot about bias assessments of the relationship between 7 negative plasma metabolites and ieu-b-5086. (C) The leave-one-out plot about sensitivity analysis of the association between 7 negative plasma metabolites and ieu-b-4981. (D) The leave-one-out plot about sensitivity analysis of the correlation b between 7 negative plasma metabolites and ieu-b-5086.

# Supplementary Tables

**Supplementary TableS1:** The causal relationship between 38 immune phenotype and sepsis in ieu-b-4981 dataset

| **exposure** | **outcome** | **method** | **nsnp** | **b** | **se** | **pval** |
| --- | --- | --- | --- | --- | --- | --- |
| CCR2 on myeloid DC | ieu-b-4981 | ivw | 9 | -0.42 | 0.18 | 0.02 |
| CD11c+ CD62L- monocyte %monocyte | ieu-b-4981 | ivw | 11 | -0.31 | 0.16 | 0.04 |
| CD19 on IgD- CD38br | ieu-b-4981 | ivw | 8 | -0.57 | 0.23 | 0.01 |
| CD19 on IgD+ | ieu-b-4981 | ivw | 13 | -0.48 | 0.17 | 0.01 |
| CD20- CD38- %lymphocyte | ieu-b-4981 | ivw | 8 | 0.58 | 0.30 | 0.04 |
| CD25 on activated Treg | ieu-b-4981 | ivw | 7 | 0.75 | 0.29 | 0.01 |
| CD25 on CD39+ CD4 Treg | ieu-b-4981 | ivw | 8 | 0.60 | 0.25 | 0.01 |
| CD25 on IgD- CD38br | ieu-b-4981 | ivw | 9 | -0.50 | 0.24 | 0.03 |
| CD27 on memory B cell | ieu-b-4981 | ivw | 16 | 0.22 | 0.11 | 0.04 |
| CD27 on sw mem | ieu-b-4981 | ivw | 19 | 0.21 | 0.10 | 0.03 |
| CD28 on CD45RA+ CD4+ | ieu-b-4981 | ivw | 11 | -0.31 | 0.15 | 0.04 |
| CD28+ CD45RA- CD8br %CD8br | ieu-b-4981 | ivw | 11 | 0.33 | 0.14 | 0.02 |
| CD3 on CD39+ CD8br | ieu-b-4981 | ivw | 9 | -0.40 | 0.17 | 0.02 |
| CD3 on HLA DR+ CD4+ | ieu-b-4981 | ivw | 18 | -0.30 | 0.12 | 0.01 |
| CD4+ %T cell | ieu-b-4981 | ivw | 8 | 0.68 | 0.26 | 0.01 |
| CD45 on CD8br | ieu-b-4981 | ivw | 7 | -0.46 | 0.22 | 0.04 |
| CD45RA- CD4+ %CD4+ | ieu-b-4981 | ivw | 14 | 0.35 | 0.17 | 0.04 |
| CD86 on myeloid DC | ieu-b-4981 | ivw | 11 | 0.40 | 0.14 | 0.01 |
| CD8br AC | ieu-b-4981 | ivw | 13 | -0.32 | 0.16 | 0.04 |
| CD8br NKT AC | ieu-b-4981 | ivw | 15 | -0.37 | 0.17 | 0.03 |
| DC AC | ieu-b-4981 | ivw | 10 | 0.41 | 0.14 | 0.00 |
| FSC-A on B cell | ieu-b-4981 | ivw | 5 | -0.58 | 0.28 | 0.04 |
| FSC-A on lymphocyte | ieu-b-4981 | ivw | 6 | 0.73 | 0.29 | 0.01 |
| HLA DR on HLA DR+ NK | ieu-b-4981 | ivw | 15 | -0.30 | 0.11 | 0.01 |
| IgD- CD38- %B cell | ieu-b-4981 | ivw | 8 | 0.48 | 0.24 | 0.04 |
| IgD- CD38br AC | ieu-b-4981 | ivw | 7 | -0.81 | 0.25 | 0.00 |
| IgD+ CD24+ %B cell | ieu-b-4981 | ivw | 10 | -0.40 | 0.18 | 0.03 |
| Leukocyte AC | ieu-b-4981 | ivw | 11 | -0.54 | 0.20 | 0.01 |
| Naive CD8br AC | ieu-b-4981 | ivw | 15 | -0.59 | 0.24 | 0.02 |
| Naive DN (CD4-CD8-) %DN | ieu-b-4981 | ivw | 10 | 0.46 | 0.22 | 0.04 |
| SSC-A on B cell | ieu-b-4981 | ivw | 8 | -0.47 | 0.21 | 0.02 |
| SSC-A on HLA DR+ CD8br | ieu-b-4981 | ivw | 11 | -0.43 | 0.21 | 0.04 |
| SSC-A on HLA DR+ NK | ieu-b-4981 | ivw | 14 | -0.30 | 0.13 | 0.02 |
| SSC-A on lymphocyte | ieu-b-4981 | ivw | 9 | 0.62 | 0.22 | 0.00 |
| SSC-A on plasmacytoid DC | ieu-b-4981 | ivw | 13 | 0.49 | 0.17 | 0.00 |
| Sw mem AC | ieu-b-4981 | ivw | 12 | 0.38 | 0.19 | 0.04 |
| TD CD4+ %T cell | ieu-b-4981 | ivw | 11 | 0.60 | 0.19 | 0.00 |
| Transitional AC | ieu-b-4981 | ivw | 18 | -0.36 | 0.17 | 0.04 |

*ivw: Inverse variance weighted; nSNP: non-synonymous single nucleotide polymorphism; pval: p value.

**Supplementary TableS2:** The causal relationship between 41 immune phenotype and sepsis in ieu-b-5086 dataset

| **exposure** | **outcome** | **method** | **nsnp** | **b** | **se** | **pval** |
| --- | --- | --- | --- | --- | --- | --- |
| BAFF-R on CD20- | ieu-b-5086 | ivw | 8 | -0.27 | 0.08 | 0.00 |
| BAFF-R on CD24+ CD27+ | ieu-b-5086 | ivw | 14 | -0.09 | 0.04 | 0.04 |
| BAFF-R on IgD- CD24- | ieu-b-5086 | ivw | 14 | -0.09 | 0.04 | 0.01 |
| BAFF-R on IgD- CD27- | ieu-b-5086 | ivw | 13 | -0.10 | 0.03 | 0.00 |
| BAFF-R on IgD- CD38- | ieu-b-5086 | ivw | 10 | -0.09 | 0.04 | 0.01 |
| BAFF-R on IgD- CD38br | ieu-b-5086 | ivw | 10 | -0.30 | 0.08 | 0.00 |
| BAFF-R on IgD- CD38dim | ieu-b-5086 | ivw | 12 | -0.09 | 0.04 | 0.03 |
| BAFF-R on IgD+ CD38- unsw mem | ieu-b-5086 | ivw | 15 | -0.08 | 0.03 | 0.02 |
| BAFF-R on IgD+ CD38dim | ieu-b-5086 | ivw | 14 | -0.07 | 0.04 | 0.04 |
| BAFF-R on memory B cell | ieu-b-5086 | ivw | 13 | -0.09 | 0.04 | 0.01 |
| BAFF-R on unsw mem | ieu-b-5086 | ivw | 11 | -0.09 | 0.04 | 0.02 |
| CD11b on CD14+ monocyte | ieu-b-5086 | ivw | 10 | 0.08 | 0.04 | 0.05 |
| CD19 on IgD+ CD38- naive | ieu-b-5086 | ivw | 6 | 0.20 | 0.09 | 0.03 |
| CD19 on IgD+ CD38br | ieu-b-5086 | ivw | 12 | -0.15 | 0.07 | 0.02 |
| CD19 on unsw mem | ieu-b-5086 | ivw | 12 | -0.21 | 0.09 | 0.02 |
| CD24+ CD27+ AC | ieu-b-5086 | ivw | 10 | -0.21 | 0.10 | 0.04 |
| CD25 on CD28+ CD4+ | ieu-b-5086 | ivw | 8 | 0.24 | 0.09 | 0.01 |
| CD25hi AC | ieu-b-5086 | ivw | 10 | 0.24 | 0.08 | 0.00 |
| CD28- CD25++ CD8br %T cell | ieu-b-5086 | ivw | 16 | 0.16 | 0.07 | 0.03 |
| CD28 on CD39+ secreting Treg | ieu-b-5086 | ivw | 12 | 0.11 | 0.06 | 0.04 |
| CD3 on NKT | ieu-b-5086 | ivw | 8 | -0.20 | 0.10 | 0.04 |
| CD33dim HLA DR+ CD11b+ %CD33dim HLA DR+ | ieu-b-5086 | ivw | 12 | 0.08 | 0.04 | 0.04 |
| CD4 on monocyte | ieu-b-5086 | ivw | 9 | -0.18 | 0.08 | 0.03 |
| CD4 Treg AC | ieu-b-5086 | ivw | 10 | 0.25 | 0.08 | 0.00 |
| CD45 on CD8br | ieu-b-5086 | ivw | 7 | -0.24 | 0.09 | 0.01 |
| CD45 on HLA DR+ CD4+ | ieu-b-5086 | ivw | 7 | 0.22 | 0.09 | 0.02 |
| CD8 on CD28- CD8br | ieu-b-5086 | ivw | 12 | -0.19 | 0.09 | 0.03 |
| CD8br AC | ieu-b-5086 | ivw | 13 | -0.18 | 0.08 | 0.03 |
| CD8dim NKT %lymphocyte | ieu-b-5086 | ivw | 13 | -0.13 | 0.06 | 0.03 |
| CD8dim NKT %T cell | ieu-b-5086 | ivw | 13 | -0.12 | 0.06 | 0.04 |
| EM CD4+ %T cell | ieu-b-5086 | ivw | 14 | 0.13 | 0.07 | 0.04 |
| EM CD4+ AC | ieu-b-5086 | ivw | 9 | 0.18 | 0.09 | 0.03 |
| FSC-A on HLA DR+ CD8br | ieu-b-5086 | ivw | 4 | 0.34 | 0.15 | 0.02 |
| HLA DR on CD14- CD16- | ieu-b-5086 | ivw | 13 | -0.11 | 0.05 | 0.05 |
| HLA DR on CD33dim HLA DR+ CD11b+ | ieu-b-5086 | ivw | 12 | -0.10 | 0.05 | 0.03 |
| IgD+ CD24+ %B cell | ieu-b-5086 | ivw | 10 | -0.17 | 0.08 | 0.02 |
| Naive CD4+ %CD4+ | ieu-b-5086 | ivw | 19 | -0.14 | 0.06 | 0.03 |
| SSC-A on granulocyte | ieu-b-5086 | ivw | 13 | -0.13 | 0.06 | 0.03 |
| SSC-A on HLA DR+ NK | ieu-b-5086 | ivw | 14 | -0.16 | 0.06 | 0.00 |
| SSC-A on plasmacytoid DC | ieu-b-5086 | ivw | 13 | 0.16 | 0.07 | 0.03 |
| T cell %leukocyte | ieu-b-5086 | ivw | 13 | 0.20 | 0.08 | 0.01 |

*ivw: Inverse variance weighted; nSNP: non-synonymous single nucleotide polymorphism; pval: p value.

**Supplementary TableS3:** The causal relationship between 57 plasma metabolites and sepsis in ieu-b-4981 dataset

| **exposure** | **outcome** | **method** | **nsnp** | **b** | **se** | **pval** |
| --- | --- | --- | --- | --- | --- | --- |
| Hypotaurine to taurine ratio | ieu-b-4981 | ivw | 23.00 | -0.50 | 0.25 | 0.04 |
| Bilirubin degradation product, C17H20N2O5 (1) levels | ieu-b-4981 | ivw | 24.00 | 0.39 | 0.20 | 0.04 |
| 2,2'-Methylenebis(6-tert-butyl-p-cresol) levels | ieu-b-4981 | ivw | 18.00 | 0.73 | 0.24 | 0.00 |
| Dopamine 4-sulfate levels | ieu-b-4981 | ivw | 24.00 | -0.41 | 0.21 | 0.04 |
| Linolenoylcarnitine (C18:3) levels | ieu-b-4981 | ivw | 22.00 | -0.52 | 0.21 | 0.01 |
| Bilirubin degradation product, C17H20N2O5 (2) levels | ieu-b-4981 | ivw | 22.00 | 0.37 | 0.16 | 0.02 |
| Uridine to pseudouridine ratio | ieu-b-4981 | ivw | 22.00 | -0.50 | 0.23 | 0.03 |
| Arachidonoylcarnitine (C20:4) levels | ieu-b-4981 | ivw | 38.00 | -0.41 | 0.12 | 0.00 |
| 1-(1-enyl-palmitoyl)-2-oleoyl-gpc (p-16:0/18:1) levels | ieu-b-4981 | ivw | 23.00 | -0.43 | 0.21 | 0.04 |
| 3,4-dihydroxybutyrate levels | ieu-b-4981 | ivw | 29.00 | 0.57 | 0.19 | 0.00 |
| Hypotaurine to cysteine ratio | ieu-b-4981 | ivw | 30.00 | -0.38 | 0.19 | 0.04 |
| Pristanate levels | ieu-b-4981 | ivw | 11.00 | 0.68 | 0.29 | 0.02 |
| Margarate (17:0) levels | ieu-b-4981 | ivw | 22.00 | 0.48 | 0.24 | 0.05 |
| 1-linoleoyl-2-arachidonoyl-GPC (18:2/20:4n6) levels | ieu-b-4981 | ivw | 24.00 | -0.42 | 0.16 | 0.01 |
| Dihomo-linolenate (20:3n3 or n6) levels | ieu-b-4981 | ivw | 26.00 | 0.46 | 0.21 | 0.03 |
| X-19438 levels | ieu-b-4981 | ivw | 22.00 | -0.46 | 0.22 | 0.04 |
| 1-(1-enyl-oleoyl)-GPE (p-18:1) levels | ieu-b-4981 | ivw | 14.00 | 0.65 | 0.30 | 0.03 |
| 2-hydroxyarachidate levels | ieu-b-4981 | ivw | 24.00 | -0.47 | 0.23 | 0.04 |
| 1-stearoyl-2-arachidonoyl-gpc (18:0/20:4) levels | ieu-b-4981 | ivw | 32.00 | -0.22 | 0.11 | 0.04 |
| Chiro-inositol levels | ieu-b-4981 | ivw | 15.00 | 0.67 | 0.21 | 0.00 |
| 5alpha-pregnan-3beta,20alpha-diol monosulfate (2) levels | ieu-b-4981 | ivw | 29.00 | 0.43 | 0.17 | 0.01 |
| Vanillic alcohol sulfate levels | ieu-b-4981 | ivw | 23.00 | -0.39 | 0.18 | 0.03 |
| 2R,3R-dihydroxybutyrate levels | ieu-b-4981 | ivw | 28.00 | -0.37 | 0.16 | 0.03 |
| Glutamine degradant levels | ieu-b-4981 | ivw | 25.00 | 0.57 | 0.27 | 0.03 |
| Hydroxypalmitoyl sphingomyelin (d18:1/16:0(OH)) levels | ieu-b-4981 | ivw | 31.00 | 0.34 | 0.16 | 0.04 |
| 1-methyl-5-imidazoleacetate levels | ieu-b-4981 | ivw | 27.00 | 0.42 | 0.21 | 0.04 |
| Glucuronate to androsterone glucuronide ratio | ieu-b-4981 | ivw | 22.00 | -0.31 | 0.15 | 0.04 |
| 3-methyl catechol sulfate (1) levels | ieu-b-4981 | ivw | 22.00 | -0.56 | 0.26 | 0.03 |
| 1-palmitoleoyl-2-linolenoyl-GPC (16:1/18:3) levels | ieu-b-4981 | ivw | 20.00 | 0.50 | 0.24 | 0.04 |
| Docosahexaenoylcarnitine (C22:6) levels | ieu-b-4981 | ivw | 29.00 | -0.52 | 0.18 | 0.00 |
| Arachidonate (20:4n6) to pyruvate ratio | ieu-b-4981 | ivw | 16.00 | -0.50 | 0.21 | 0.02 |
| 2-hydroxybutyrate/2-hydroxyisobutyrate levels | ieu-b-4981 | ivw | 13.00 | -0.73 | 0.30 | 0.01 |
| X-23644 levels | ieu-b-4981 | ivw | 16.00 | 0.72 | 0.32 | 0.03 |
| X-25217 levels | ieu-b-4981 | ivw | 15.00 | 0.46 | 0.23 | 0.04 |
| Taurine to glutamate ratio | ieu-b-4981 | ivw | 19.00 | 0.56 | 0.27 | 0.04 |
| X-12822 levels | ieu-b-4981 | ivw | 20.00 | -0.47 | 0.19 | 0.01 |
| phosphate to asparagine ratio | ieu-b-4981 | ivw | 24.00 | -0.51 | 0.22 | 0.02 |
| 1-methyl-4-imidazoleacetate levels | ieu-b-4981 | ivw | 27.00 | 0.33 | 0.16 | 0.04 |
| Inosine levels | ieu-b-4981 | ivw | 20.00 | 0.52 | 0.20 | 0.01 |
| X-11880 levels | ieu-b-4981 | ivw | 17.00 | -0.69 | 0.26 | 0.01 |
| 3-methyl-2-oxovalerate to 4-methyl-2-oxopentanoate ratio | ieu-b-4981 | ivw | 22.00 | -0.57 | 0.25 | 0.02 |
| Aspartate to citrulline ratio | ieu-b-4981 | ivw | 17.00 | -0.58 | 0.27 | 0.03 |
| Vanillactate levels | ieu-b-4981 | ivw | 26.00 | 0.48 | 0.23 | 0.03 |
| 5-hydroxyindole sulfate levels | ieu-b-4981 | ivw | 17.00 | -0.49 | 0.22 | 0.03 |
| Methyl-4-hydroxybenzoate sulfate levels | ieu-b-4981 | ivw | 24.00 | -0.55 | 0.22 | 0.01 |
| Choline phosphate to choline ratio | ieu-b-4981 | ivw | 21.00 | -0.82 | 0.23 | 0.00 |
| (16 or 17)-methylstearate (a19:0 or i19:0) levels | ieu-b-4981 | ivw | 18.00 | -0.75 | 0.26 | 0.00 |
| Malate levels | ieu-b-4981 | ivw | 18.00 | -0.64 | 0.22 | 0.00 |
| X-23587 levels | ieu-b-4981 | ivw | 18.00 | 0.58 | 0.27 | 0.03 |
| X-11470 levels | ieu-b-4981 | ivw | 39.00 | 0.30 | 0.12 | 0.01 |
| X-21752 levels | ieu-b-4981 | ivw | 22.00 | 0.58 | 0.21 | 0.01 |
| 1-palmitoyl-2-palmitoleoyl-gpc (16:0/16:1) levels | ieu-b-4981 | ivw | 24.00 | 0.44 | 0.22 | 0.04 |
| Docosahexaenoylcholine levels | ieu-b-4981 | ivw | 15.00 | 0.73 | 0.28 | 0.01 |
| X-12127 levels | ieu-b-4981 | ivw | 37.00 | 0.34 | 0.15 | 0.03 |
| 9,10-DiHOME levels | ieu-b-4981 | ivw | 17.00 | 0.53 | 0.25 | 0.04 |
| Cysteine s-sulfate levels | ieu-b-4981 | ivw | 15.00 | -0.68 | 0.29 | 0.02 |
| Sulfate of piperine metabolite C18H21NO3 (3) levels | ieu-b-4981 | ivw | 20.00 | 0.51 | 0.23 | 0.02 |

*ivw: Inverse variance weighted; nSNP: non-synonymous single nucleotide polymorphism; pval: p value.

**Supplementary TableS4:** The causal relationship between 63 plasma metabolites and sepsis in ieu-b-5086 dataset

| **exposure** | **outcome** | **method** | **nsnp** | **b** | **se** | **pval** |
| --- | --- | --- | --- | --- | --- | --- |
| Octadecanedioylcarnitine (C18-DC) levels | ieu-b-5086 | ivw | 23 | -0.12 | 0.06 | 0.05 |
| Bilirubin degradation product, C17H20N2O5 (1) levels | ieu-b-5086 | ivw | 24 | 0.15 | 0.07 | 0.04 |
| Methionine sulfone levels | ieu-b-5086 | ivw | 35 | 0.16 | 0.06 | 0.01 |
| Trans 3,4-methyleneheptanoate levels | ieu-b-5086 | ivw | 13 | -0.37 | 0.12 | 0.00 |
| 2-furoylcarnitine levels | ieu-b-5086 | ivw | 18 | 0.20 | 0.10 | 0.04 |
| X-26054 levels | ieu-b-5086 | ivw | 25 | 0.13 | 0.06 | 0.03 |
| Erythritol levels in elite athletes | ieu-b-5086 | ivw | 25 | 0.20 | 0.08 | 0.02 |
| 1-(1-enyl-palmitoyl)-2-oleoyl-gpc (p-16:0/18:1) levels | ieu-b-5086 | ivw | 23 | -0.29 | 0.09 | 0.00 |
| Phosphate levels (UKB data field 30810) | ieu-b-5086 | ivw | 21 | -0.23 | 0.10 | 0.02 |
| 3-ethylcatechol sulfate (1) levels | ieu-b-5086 | ivw | 19 | 0.20 | 0.10 | 0.04 |
| X-22520 levels | ieu-b-5086 | ivw | 17 | 0.20 | 0.09 | 0.03 |
| Vanillic acid glycine levels | ieu-b-5086 | ivw | 22 | 0.18 | 0.09 | 0.04 |
| 1-linoleoyl-2-arachidonoyl-GPC (18:2/20:4n6) levels | ieu-b-5086 | ivw | 24 | -0.22 | 0.08 | 0.00 |
| Octadecadienedioate (C18:2-DC) levels | ieu-b-5086 | ivw | 30 | -0.11 | 0.06 | 0.05 |
| Glycerol to sulfate ratio | ieu-b-5086 | ivw | 21 | 0.28 | 0.13 | 0.03 |
| Linoleoyl-arachidonoyl-glycerol (18:2/20:4) [2] levels | ieu-b-5086 | ivw | 34 | -0.14 | 0.06 | 0.02 |
| 1-stearoyl-2-arachidonoyl-gpc (18:0/20:4) levels | ieu-b-5086 | ivw | 32 | -0.15 | 0.05 | 0.00 |
| 1-stearoyl-gpc (18:0) levels | ieu-b-5086 | ivw | 28 | -0.23 | 0.09 | 0.01 |
| Oleoylcholine levels | ieu-b-5086 | ivw | 18 | 0.24 | 0.11 | 0.03 |
| Adenosine 5'-monophosphate (AMP) to EDTA ratio | ieu-b-5086 | ivw | 14 | 0.27 | 0.13 | 0.04 |
| Glycosyl ceramide (d18:1/20:0, d16:1/22:0) levels | ieu-b-5086 | ivw | 33 | -0.14 | 0.07 | 0.04 |
| Cysteine levels | ieu-b-5086 | ivw | 12 | 0.28 | 0.13 | 0.04 |
| Palmitoleoylcarnitine (C16:1) levels | ieu-b-5086 | ivw | 16 | 0.25 | 0.12 | 0.03 |
| Glutamine degradant levels | ieu-b-5086 | ivw | 25 | 0.21 | 0.09 | 0.02 |
| 2-hydroxyhippurate (salicylurate) levels | ieu-b-5086 | ivw | 22 | 0.22 | 0.10 | 0.03 |
| Proline to glutamate ratio | ieu-b-5086 | ivw | 21 | -0.20 | 0.10 | 0.03 |
| X-18888 levels | ieu-b-5086 | ivw | 21 | -0.29 | 0.09 | 0.00 |
| Hydroxypalmitoyl sphingomyelin (d18:1/16:0(OH)) levels | ieu-b-5086 | ivw | 31 | 0.18 | 0.07 | 0.01 |
| Salicylate to taurocholate ratio | ieu-b-5086 | ivw | 25 | -0.28 | 0.09 | 0.00 |
| X-15523 levels | ieu-b-5086 | ivw | 54 | -0.15 | 0.06 | 0.01 |
| 1H-indole-7-acetic acid levels | ieu-b-5086 | ivw | 29 | 0.28 | 0.07 | 0.00 |
| 1-myristoyl-2-arachidonoyl-GPC (14:0/20:4) levels | ieu-b-5086 | ivw | 22 | -0.18 | 0.07 | 0.01 |
| Imidazole propionate levels | ieu-b-5086 | ivw | 24 | 0.20 | 0.09 | 0.03 |
| Alpha-ketoglutarate to alanine ratio | ieu-b-5086 | ivw | 19 | -0.22 | 0.10 | 0.03 |
| Adenosine 5'-monophosphate (AMP) to threonine ratio | ieu-b-5086 | ivw | 21 | 0.23 | 0.10 | 0.03 |
| N-acetylmethionine levels | ieu-b-5086 | ivw | 10 | 0.30 | 0.15 | 0.04 |
| Docosahexaenoylcarnitine (C22:6) levels | ieu-b-5086 | ivw | 29 | -0.18 | 0.08 | 0.03 |
| Adenosine 5'-diphosphate (ADP) to aspartate ratio | ieu-b-5086 | ivw | 23 | -0.22 | 0.08 | 0.01 |
| Glycerol to carnitine ratio | ieu-b-5086 | ivw | 26 | 0.24 | 0.10 | 0.02 |
| Glycodeoxycholate levels | ieu-b-5086 | ivw | 16 | 0.24 | 0.11 | 0.03 |
| Dihomo-linoleate (20:2n6) levels | ieu-b-5086 | ivw | 17 | 0.23 | 0.12 | 0.05 |
| phosphate to asparagine ratio | ieu-b-5086 | ivw | 24 | -0.20 | 0.09 | 0.04 |
| Inosine levels | ieu-b-5086 | ivw | 20 | 0.16 | 0.08 | 0.05 |
| Lignoceroyl sphingomyelin (d18:1/24:0) levels | ieu-b-5086 | ivw | 23 | 0.34 | 0.11 | 0.00 |
| 4-acetylphenol sulfate levels | ieu-b-5086 | ivw | 17 | -0.22 | 0.11 | 0.04 |
| Sphingomyelin (d18:1/18: 1, d18:2/18:0) levels | ieu-b-5086 | ivw | 28 | -0.22 | 0.08 | 0.01 |
| Methyl-4-hydroxybenzoate sulfate levels | ieu-b-5086 | ivw | 24 | -0.28 | 0.10 | 0.00 |
| Phenylpyruvate levels | ieu-b-5086 | ivw | 28 | 0.17 | 0.08 | 0.04 |
| Malate levels | ieu-b-5086 | ivw | 18 | -0.20 | 0.09 | 0.03 |
| X-23587 levels | ieu-b-5086 | ivw | 18 | 0.25 | 0.11 | 0.02 |
| 9,10-DiHOME levels | ieu-b-5086 | ivw | 17 | 0.23 | 0.11 | 0.03 |
| Cholesterol to linoleoyl-arachidonoyl-glycerol (18:2 to 20:4) [2] ratio | ieu-b-5086 | ivw | 30 | 0.16 | 0.07 | 0.03 |
| Caffeic acid sulfate levels | ieu-b-5086 | ivw | 23 | -0.19 | 0.09 | 0.04 |
| 1-arachidonoyl-GPE (20:4n6) levels | ieu-b-5086 | ivw | 32 | -0.13 | 0.06 | 0.04 |
| 3-methylhistidine levels | ieu-b-5086 | ivw | 27 | -0.23 | 0.10 | 0.02 |
| 5-oxoproline levels | ieu-b-5086 | ivw | 25 | 0.15 | 0.06 | 0.01 |
| N2, n2-dimethylguanosine levels | ieu-b-5086 | ivw | 15 | -0.31 | 0.13 | 0.02 |
| N-formylphenylalanine levels | ieu-b-5086 | ivw | 32 | -0.16 | 0.08 | 0.04 |
| X-12847 levels | ieu-b-5086 | ivw | 16 | -0.21 | 0.11 | 0.04 |
| 5-hydroxylysine levels | ieu-b-5086 | ivw | 27 | 0.16 | 0.07 | 0.03 |
| Dihomo-linolenoyl-choline levels | ieu-b-5086 | ivw | 26 | -0.18 | 0.08 | 0.03 |
| Hypotaurine levels | ieu-b-5086 | ivw | 25 | -0.35 | 0.09 | 0.00 |
| Metabolonic lactone sulfate levels | ieu-b-5086 | ivw | 37 | -0.10 | 0.04 | 0.01 |

*ivw: Inverse variance weighted; nSNP: non-synonymous single nucleotide polymorphism; pval: p value.
